# Socioeconomic status and the 25 × 25 risk factors as determinants of premature mortality: a multicohort study and meta-analysis of 1·7 million men and women

**DOI:** 10.1016/S0140-6736(16)32380-7

**Published:** 2017-03-25

**Authors:** Silvia Stringhini, Cristian Carmeli, Markus Jokela, Mauricio Avendaño, Peter Muennig, Florence Guida, Fulvio Ricceri, Angelo d'Errico, Henrique Barros, Murielle Bochud, Marc Chadeau-Hyam, Françoise Clavel-Chapelon, Giuseppe Costa, Cyrille Delpierre, Silvia Fraga, Marcel Goldberg, Graham G Giles, Vittorio Krogh, Michelle Kelly-Irving, Richard Layte, Aurélie M Lasserre, Michael G Marmot, Martin Preisig, Martin J Shipley, Peter Vollenweider, Marie Zins, Ichiro Kawachi, Andrew Steptoe, Johan P Mackenbach, Paolo Vineis, Mika Kivimäki, LIFEPATH consortium, Harri Alenius, Mauricio Avendano, Henrique Barros, Murielle Bochud, Cristian Carmeli, Luca Carra, Raphaele Castagné, Marc Chadeau-Hyam, Françoise Clavel-Chapelon, Giuseppe Costa, Emilie Courtin, Cyrille Delpierre, Angelo D'Errico, Pierre-Antoine Dugué, Paul Elliott, Silvia Fraga, Valérie Gares, Graham Giles, Marcel Goldberg, Dario Greco, Allison Hodge, Michelle Kelly Irving, Piia Karisola, Mika Kivimäki, Vittorio Krogh, Thierry Lang, Richard Layte, Benoit Lepage, Johan Mackenbach, Michael Marmot, Cathal McCrory, Roger Milne, Peter Muennig, Wilma Nusselder, Salvatore Panico, Dusan Petrovic, Silvia Polidoro, Martin Preisig, Olli Raitakari, Ana Isabel Ribeiro, Ana Isabel Ribeiro, Fulvio Ricceri, Oliver Robinson, Jose Rubio Valverde, Carlotta Sacerdote, Roberto Satolli, Gianluca Severi, Martin J Shipley, Silvia Stringhini, Rosario Tumino, Paolo Vineis, Peter Vollenweider, Marie Zins

**Affiliations:** a Institute of Social and Preventive Medicine and Departments of Psychiatry and Internal Medicine, Lausanne University Hospital, Lausanne, Switzerland; b Institute of Behavioural Sciences, University of Helsinki, Helsinki, Finland; c Department of Global Health and Social Medicine, King's College London, London, UK; d Harvard T H Chan School of Public Health, Boston MA, USA; e Global Research Analytics for Population Health, Health Policy and Management, Columbia University, New York, NY, USA; f MRC-PHE Centre for Environment and Health, School of Public Health, Department of Epidemiology and Biostatistics, Imperial College London, London, UK; g Epidemiology Unit, ASL TO3 Piedmont Region, Grugliasco, Italy; h EPIUnit–Institute of Public Health, University of Porto, Porto, Portugal; i Department of Clinical Epidemiology, Predictive Medicine and Public Health, University of Porto Medical School, Porto, Portugal; j Center for Research in Epidemiology and Population Health, INSERM U1018, Villejuif, France; k Department of Biological and Clinical Sciences, Universtiy of Turin, Turin, Italy; l INSERM, UMR1027, Toulouse, France; m Université Toulouse III Paul-Sabatier, UMR1027, Toulouse, France; n Population-based Epidemiological Cohorts Unit, INSERM UMS 11, Villejuif, France; o Paris Descartes University, Paris, France; p Cancer Epidemiology Centre, Cancer Council Victoria, Melbourne, VIC, Australia; q Epidemiology and Prevention Unit, Fondazione IRCCS Istituto Nazionale dei Tumori, Milan, Italy; r Department of Sociology, Trinity College Dublin, Dublin, Ireland; s University College London, Department of Epidemiology and Public Health, London, UK; t Department of Public Health, Erasmus University Medical Center, Rotterdam, Netherlands; u Clinicum, Faculty of Medicine, University of Helsinki, Finland

## Abstract

**Background:**

In 2011, WHO member states signed up to the 25 × 25 initiative, a plan to cut mortality due to non-communicable diseases by 25% by 2025. However, socioeconomic factors influencing non-communicable diseases have not been included in the plan. In this study, we aimed to compare the contribution of socioeconomic status to mortality and years-of-life-lost with that of the 25 × 25 conventional risk factors.

**Methods:**

We did a multicohort study and meta-analysis with individual-level data from 48 independent prospective cohort studies with information about socioeconomic status, indexed by occupational position, 25 × 25 risk factors (high alcohol intake, physical inactivity, current smoking, hypertension, diabetes, and obesity), and mortality, for a total population of 1 751 479 (54% women) from seven high-income WHO member countries. We estimated the association of socioeconomic status and the 25 × 25 risk factors with all-cause mortality and cause-specific mortality by calculating minimally adjusted and mutually adjusted hazard ratios [HR] and 95% CIs. We also estimated the population attributable fraction and the years of life lost due to suboptimal risk factors.

**Findings:**

During 26·6 million person-years at risk (mean follow-up 13·3 years [SD 6·4 years]), 310 277 participants died. HR for the 25 × 25 risk factors and mortality varied between 1·04 (95% CI 0·98–1·11) for obesity in men and 2 ·17 (2·06–2·29) for current smoking in men. Participants with low socioeconomic status had greater mortality compared with those with high socioeconomic status (HR 1·42, 95% CI 1·38–1·45 for men; 1·34, 1·28–1·39 for women); this association remained significant in mutually adjusted models that included the 25 × 25 factors (HR 1·26, 1·21–1·32, men and women combined). The population attributable fraction was highest for smoking, followed by physical inactivity then *s*ocioeconomic status. Low socioeconomic status was associated with a 2·1-year reduction in life expectancy between ages 40 and 85 years, the corresponding years-of-life-lost were 0·5 years for high alcohol intake, 0·7 years for obesity, 3·9 years for diabetes, 1·6 years for hypertension, 2·4 years for physical inactivity, and 4·8 years for current smoking.

**Interpretation:**

Socioeconomic circumstances, in addition to the 25 × 25 factors, should be targeted by local and global health strategies and health risk surveillance to reduce mortality.

**Funding:**

European Commission, Swiss State Secretariat for Education, Swiss National Science Foundation, the Medical Research Council, NordForsk, Portuguese Foundation for Science and Technology.

## Introduction

The 2013–20 World Health Organization (WHO) Global Action Plan for the Prevention and Control of Non-Communicable Diseases (NCDs) targets seven major risk factors, comprising the harmful use of alcohol, insufficient physical activity, current tobacco use, raised blood pressure, intake of salt or sodium, diabetes, and obesity (referred to as the 25 × 25 risk factors), with the overall aim of reducing premature mortality from non-communicable diseases by 25% by 2025.^1^ Similarly, the Global Burden of Disease (GBD) Collaboration, the largest study monitoring health changes globally, performs an annual risk assessment of the burden of disease and injury attributable to 67 risk factors in 21 world-regions.^2^ Despite the fact that low socioeconomic status is one of the strongest predictors of morbidity and premature mortality worldwide,^3^, ^4^, ^5^, ^6^ poor socioeconomic circumstances are not considered modifiable risk factors in these important global health strategies.

Socioeconomic circumstances and their consequences are modifiable by policies at the local, national, and international levels,^7^, ^8^, ^9^ as are risk factors targeted by existing global health strategies. Evidence also suggests that the burden of most 25 × 25 risk factors is concentrated in lower socioeconomic groups worldwide.^10^, ^11^ Interventions to reduce premature mortality attributable to the 25 × 25 and other risk factors might therefore benefit from greater focus on socioeconomic adversity so that the preventive toolkit for addressing NCDs can be expanded. To examine this hypothesis, we collated individual-level data from 48 independent prospective cohort studies from Europe, the USA, and Australia and aimed to determine the population attributable fraction (PAF) and years of life lost (YLLs) due to low socioeconomic status and compared these with mortality and YLLs attributable to the 25 × 25 risk factors.

**Research in context**

**Evidence before this study**

Low socioeconomic status is one of the strongest predictors of morbidity and premature mortality worldwide. However, global health strategies do not consider poor socioeconomic circumstances as modifiable risk factors. The WHO Global Action Plan for the Prevention and Control of Non-Communicable Diseases, for example, targets seven major health risk factors, including insufficient physical activity, current tobacco use. and raised blood pressure, for reducing premature mortality from non-communicable diseases by 25% by 2025. Low socioeconomic status is not included among the 25 × 25 risk factors.

**Added value of this study**

We used data from more than 1·7 million individuals in 48 independent cohort studies from seven countries, and found that the independent association between socioeconomic status and mortality is comparable in strength and consistency to those of six 25 × 25 risk factors (tobacco use, alcohol consumption, insufficient physical activity, raised blood pressure, obesity, diabetes). Our study is one of the largest studies to date to examine the association between socioeconomic status and premature mortality and the first large-scale investigation to directly compare the importance of socioeconomic circumstances as determinants of health with six major risk factors targeted in global health strategies for the reduction of premature mortality.

**Implications of all the available evidence**

By showing comparable health impact of low socioeconomic status to that of major risk factors, our study suggests that socioeconomic adversity should be included as a modifiable risk factor in local and global health strategies, policies, and health-risk surveillance.

## Methods

### Study population

This study is part of an EC Horizon 2020 consortium, the Lifepath project, which includes ten cohort studies. We have complemented those data with publicly available data from 38 additional cohort studies from the Inter-University Consortium for Political and Social Research and the UK Data Service. Our analyses were based on participants whose occupational position was assessed at baseline between 1965 and 2009, dependent on the study (appendix). The 48 studies comprised a total population of 1 751 479 men and women from seven WHO member countries (UK, France, Switzerland, Portugal, Italy, USA, Australia). All studies included baseline data for socioeconomic status and a mortality follow-up of a minimum of 3 years. Each study was approved by the relevant local or national ethics committees and all participants gave informed consent to participate. We assessed the quality of included studies using the Cochrane Risk of Bias Tool for cohort studies.^12^ We analysed a selection of exposed and non-exposed groups, assessment of exposure, exclusion of the outcome of interest at study baseline, adjustment for confounding variables, assessment of confounding variables, assessment of outcome, and adequacy of the follow-up. Two reviewers (SS and MK) independently assessed the studies. The quality of the study was judged as high if all domains were assessed favourably (appendix).

### Definitions and data collection

Our measure of socioeconomic status is a social class measure based on an individual's last known occupational title at study enrolment, coded into the European Socio-economic Classification (ESEC). This variable was predefined and harmonised across the study cohorts before statistical analyses were done. Occupational position was categorised as high (higher professionals and managers, higher clerical, services, and sales workers [ESEC class 1, 2, and 3]), intermediate (small employers and self-employed, farmers, lower supervisors, and technicians [ESEC class 4, 5, and 6]), or low (lower clerical, services and sales workers, skilled workers, and semi-skilled and unskilled workers [ESEC class 7, 8, and 9]). For one study (E3N), occupational position was current occupation 2 years after baseline. We used ESEC as a classification because it eliminates the need to adjust for differences in earnings and standards of living across different national contexts. We used individual's occupational class only because most cohorts did not collect information about partner's occupation. This decision could have led to some misclassification of socioeconomic status particularly for older women with low labour force participation rates.

Each 25 × 25 risk factor comprised two or three categories to allow a balanced comparison with socioeconomic status, which was grouped into three categories (appendix). Self-reported smoking was categorised into current smoker, former smoker, and never smoked. Alcohol consumption was measured in alcohol units per week and participants were categorised as abstainers (0 units per week), moderate (1–21 units per week for men, 1–14 per week for women), or heavy (>21 units per week for men, >14 per week for women) drinkers. Although physical activity was measured with different questions in each study, a dichotomised variable indicating the presence or absence of physical activity was defined (appendix). Body-mass index (BMI) was categorised as normal (18·5–<25 kg/m^2^), overweight (25–<30 kg/m^2^), or obese (≥30 kg/m^2^). Hypertension was defined as the presence of at least one of the following conditions: systolic blood pressure more than 140 mm Hg, diastolic blood pressure more than 90 mm Hg, current intake of anti-hypertensive medication, or self-reported hypertension. Diabetes was defined as the presence of at least one of the following conditions: fasting glucose more than 7 mmol/L, 2 h post-load glucose above 11·1 mmol/L, glycated haemoglobin A1c more than 6·5%, or self-reported diabetes. Data for salt intake were only available from less than a third of the cohort studies; we therefore omitted this risk factor from our analysis.

We considered age, sex, race or ethnicity, and marital status as potential confounders. Race or ethnicity was categorised as white and non-white individuals. Marital status was categorised as married or cohabiting versus living alone.

Participants were linked to national mortality registries that provided information about vital status with the exception of the COLAUS study in which vital status was ascertained through active follow-up. Mean follow-up for mortality ranged between 3·2 years in the National Health Interview Survey 2009, and 27·0 years in men and 29·5 years in women of the Alameda County Study 1965, with a mean across cohorts of 13·3 years [SD 6·4 years]. All-cause mortality, cancer mortality, cardiovascular disease mortality, and mortality from other causes of death were examined separately. We focus on cancer and cardiovascular disease as these diseases are the most common causes of death in our samples. We used codes from the International Classification of Diseases, 10th Revision (ICD-10) to define cancer (C00–C97) and cardiovascular disease (I00–I99) mortality. Other causes of death include all remaining deaths not classified as cancer or cardiovascular disease.

### Statistical analysis

Analyses were first performed separately in each study; estimates were subsequently combined in a meta-analytical framework. In study-specific analyses, we considered the maximum number of participants without missing values for each exposure. To estimate the association between risk factors and mortality, hazard ratios (HR) and 95% CIs were generated using flexible parametric survival models on the cumulative hazards scale,^13^ which, in addition to the HRs, allow direct estimation of the conditional cumulative hazard function. Within these models, we used restricted cubic splines with 0 to 4 (depending on the cohort) internal knots to model the baseline hazard using age as the timescale. Separate models were fitted for men and women and included marital status and race or ethnicity (minimally adjusted models). To check for the proportional hazard assumption, we performed tests based on Schoenfeld residuals and inspected log-log plots of Kaplan-Meier curves. Age stratification in 5-year intervals was conducted in all cohorts as a sensitivity analysis to adjust for age calendar effects (results not shown).

In further analyses combining men and women, we examined the association of socioeconomic status with cause-specific mortality before and after adjustment for the 25 × 25 risk factors. The mutually adjusted models included age, sex, race or ethnicity, marital status, socioeconomic status, and all 25 × 25 risk factors as independent variables with total mortality and deaths from cardiovascular disease, cancer, and other causes as outcomes. To enable balanced comparisons between socioeconomic status and 25 × 25 risk factors as predictors of cause-specific mortality, these analyses were restricted to a subgroup of participants with complete data for socioeconomic status and the 25 × 25 risk factors.

To examine whether the association between socioeconomic status and mortality is attributable to the higher prevalence of the 25 × 25 risk factors among low socioeconomic status individuals, we repeated the analyses in a subgroup of participants without any 25 × 25 risk factors. Analyses were also repeated specifically focusing on premature mortality (<70 years) and by restricting the population to cohorts in which height and weight as well as blood pressure were measured objectively using standard procedures.

To further evaluate the effects of socioeconomic status and the 25 × 25 risk factors on mortality, we computed the population attributable fraction. The population attributable fraction is based on the HR and the proportion of participants exposed assuming the association between exposure and outcome is causal.^14^ The variance of population attributable fraction was estimated via bootstrapping using 1000 independent replications. The proportion of participants exposed (prevalence) was calculated as the mean prevalence across all cohorts for each risk factor.

YLLs were calculated as the difference of the areas under the survival curves (from age 40 years to 85 years) comparing the population exposed to a given risk factor with the reference population with no exposure. Area under the curve was computed via numerical integration with a spline-based method. Life expectancies were estimated conditional on survival to age 40 years. In view of the truncation at age 85 years, the theoretical maximal life expectancy at 40 years old is 45 years. Variance of YLLs was estimated via bootstrapping using 1000 independent replications.

Study-specific HRs, PAF, and YLLs estimates were meta-analysed using the Hartung-Knapp random-effects method.^15^ To assess heterogeneity between cohorts, we computed *I*^2^ and τ^2^ statistics; *I*^2^ to assess heterogeneity attributable to variation in the true association and τ^2^ to measure the inter-cohort variance. To account for τ^2^ in the uncertainty around the pooled estimates, we further calculated 95% prediction intervals for hazard ratios.^16^

### Role of the funding source

The funding sources had no role in the study design; in the collection, analysis, and interpretation of data; in the writing of the report; or in the decision to submit the paper for publication. CC and MJ had full access to the datasets. SS, PV, and MK had final responsibility for the decision to submit for publication.

## Results

48 studies were included (appendix). After excluding 27 392 (1·5%) of 1 778 871 participants who had missing data for the covariates or mortality, 1 751 479 participants were included in the analysis (appendix). Mean age at study entry was 47·8 years (SD 14·8) and 54% of participants were women. The proportion of participants with low occupational position ranged from 6·9% to 66·9% across studies (mean 41·4% [SD 12·5] for men and 27·1% [SD 14·9] for women). The proportion of people with a high occupational position varied between 5·9% and 84·8% (mean 32·5% [SD 11·7] men and 26·1% [SD 12·3] women). Age stratification revealed no age calendar effects (data not shown).

**Figure 1:**
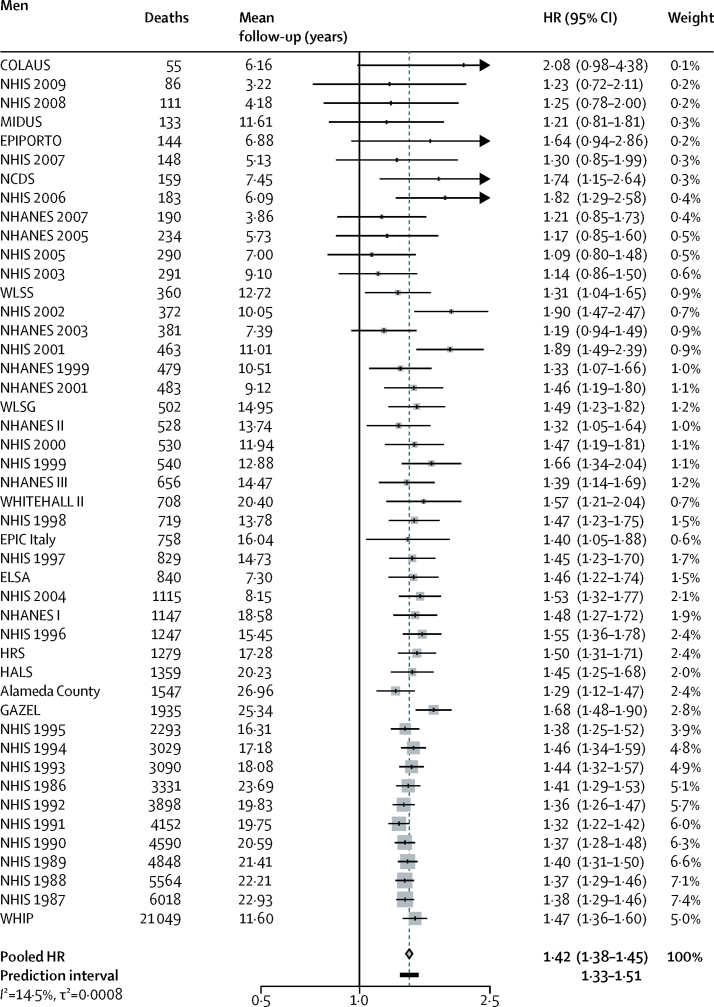
Mortality for low versus high occupational position in men in 46 cohort studies HRs are adjusted for age, marital status, and race or ethnicity. Pooled HR is represented with a grey diamond and the 95% prediction interval with a black bar. *I*^2^ statistic is the percentage of between study heterogeneity; τ^2^ statistic measures the inter-study variance. The prediction interval provides a predicted range for the true association between occupational position and mortality. HR=hazard ratio.

**Figure 2:**
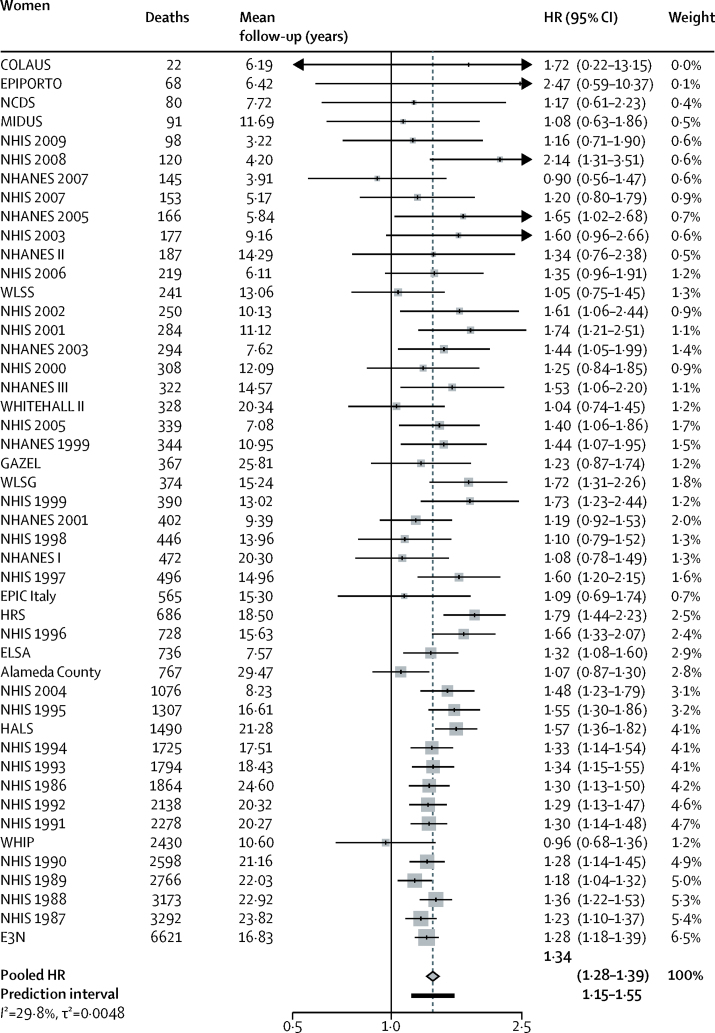
Mortality for low versus high occupational position in women in 47 cohort studies HRs are adjusted for age, marital status, and race or ethnicity. Pooled HR is represented with a grey diamond and the 95% prediction interval with a black bar. The prediction interval provides a predicted range for the true association between occupational position and mortality. HR=hazard ratio.

During 12 025 208 person-years at risk for men, 161 524 men died; during 14 580 862 person-years at risk for women, a total of 148 753 women died (mean follow-up for men and women 13·3 years [SD 3·4]). In men, 43 765 (15·2% of total) with low occupational position died and 17 160 (11·5%) with high occupational position died. In women, 11 835 (9·4% of total) with low occupational position died and 8292 (6·8%) with high occupational position died. Participants with low occupational position had a higher mortality risk than did those with high occupational position, in both men (HR 1·42, 95% CI 1·38–1·45; figure 1) and women (1·34, 1·28–1·39; figure 2). Participants with intermediate occupational position had a higher mortality risk compared with participants with high occupational position (meta-analytic HR 1·21, 95% CI 1·18–1·24 for men and 1·17, 1·12–1·22 for women). A graded association between occupational position and mortality was observed in both men and women (HR for one unit decrease in SES 1·19, 95% CI 1·17–1·20 in men and 1·15, 1·13–1·18 in women, p<0·0001 for both). Heterogeneity in study-specific estimates was low for men (*I*^2^=14·5% [0–41%], p=0·2034, τ^2^=0·0008) and moderate for women (*I*^2^=29·8% [0–51·2%], p=0·0309, τ^2^=0·0048).

**Figure 3:**
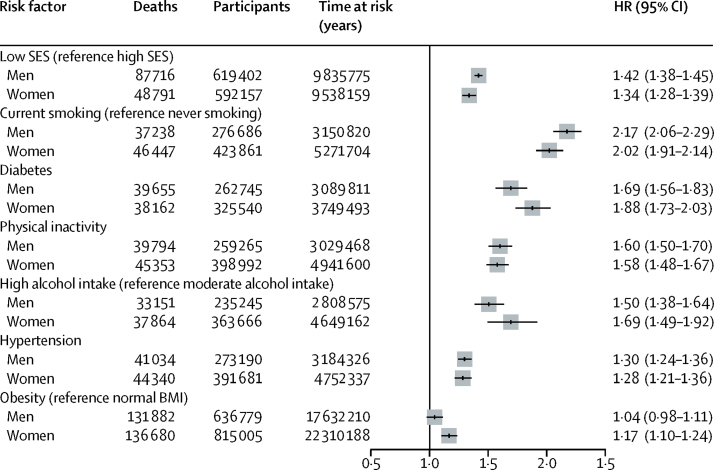
Pooled hazard ratios of socioeconomic status and 25 × 25 risk factors for mortality HRs are adjusted for age, marital status, and race or ethnicity. SES=socioeconomic status. BMI=body-mass index.

**Figure 4:**
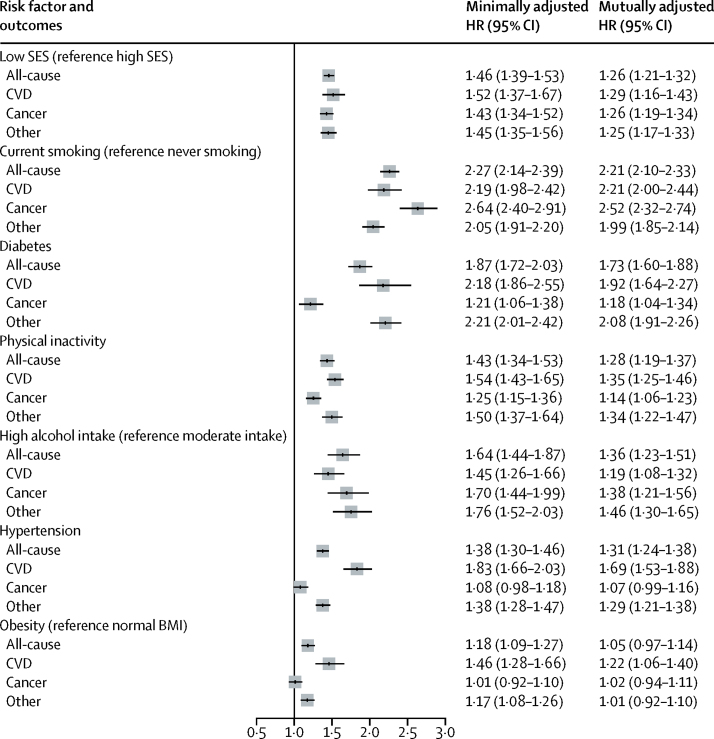
Pooled hazard ratios of socioeconomic status and 25 × 25 risk factors for all-cause mortality and cause-specific mortality The minimally adjusted models were only adjusted for sex, age, and race or ethnicity; in the mutually adjusted models, SES and the 25 × 25 risk factors are mutually adjusted. BMI=body-mass index. CVD=cardiovascular disease. SES=socioeconomic status.

Figure 3 shows mortality associated with the 25 × 25 risk factors (minimally adjusted models). The greatest increases in mortality associated with the 25 × 25 risk factors were for current smoking and diabetes, although physical inactivity, high alcohol intake, and hypertension were also associated (figure 3). The effect of low occupational position appeared greater than that of hypertension or obesity (figure 3); the effect of low occupational position on mortality was greater than that of obesity even when the obesity analysis was restricted to cohorts with a mean follow-up more than 10 years (>10 years; HR 1·12, 95% CI 1·05–1·21 for men and 1·24, 1·18–1·31 for women). 33 of 48 studies had complete data for occupational position and all 25 × 25 risk factors and had cause-specific mortality data, for a total of 275 973 participants with 21 923 deaths during the follow-up (figure 4). The association between low socioeconomic status and mortality was consistent across causes of death and remained significant in the minimally adjusted models and the mutually adjusted models (figure 4). The highest minimally adjusted HR was current smoking (figure 4).

**Figure 5:**
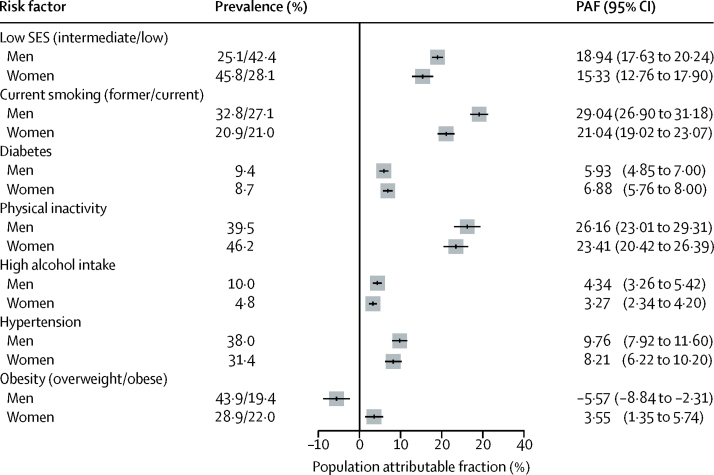
Population attributable fraction for socioeconomic status and 25 × 25 risk factors Calculations assume risk in the population at the level of the least exposed group. SES=socioeconomic status. PAF=population attributable fraction.

We assessed the PAF for socioeconomic status and the 25 × 25 risk factors, assuming the associations with mortality are causal and that the risk could be reduced to the level of the most favourable category for each factor (figure 5). We estimated the achievable reduction in mortality during the follow-up period should the death risk in the whole population equate that of high occupational position or the reference group for each of the 25 × 25 risk factors. The PAF for low SES was 18·94% (95% CI 17·63–20·24) for men and 15·33% (12·76–17·90) for women. The highest PAF was for smoking for men (29·04%, 26·90–31·18) and for physical inactivity for women (23·41%, 20·42–26·39).

**Figure 6:**
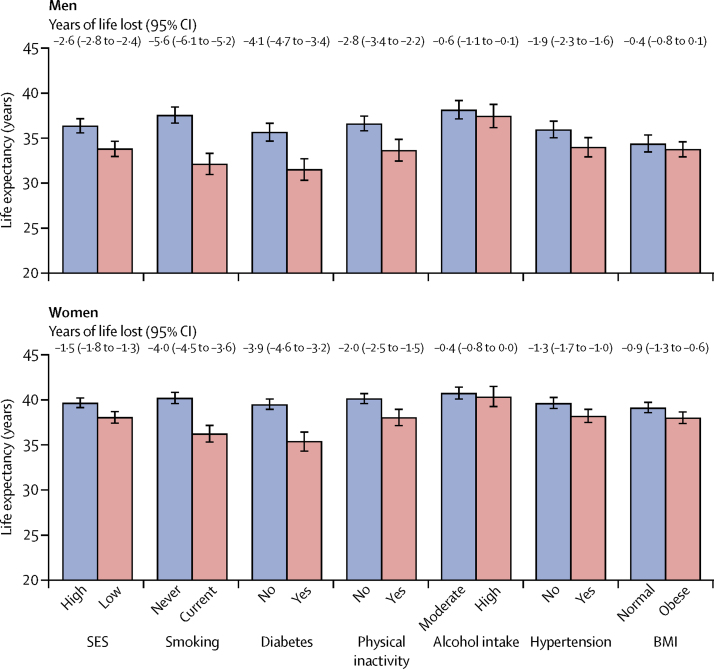
Life expectancy from age 40 years to 85 years and years of life lost due to low socioeconomic status and 25 × 25 risk factors SES=socioeconomic status. BMI=body-mass index.

In men and women combined, partial life expectancy at 40 years was reduced by more than 2 years because of low socioeconomic status (figure 6). All other 25 × 25 factors assessed were associated with decreased life expectancy, apart from BMI (figure 6).

Additional sensitivity analyses including only western European cohorts, restricting the analysis to premature mortality (<70 years), to a subset of participants without the 25 × 25 risk factors (HR for low SES *vs* high SES 1·26, 95% CI 1·12–1·42), and to high quality studies or to cohorts with height and weight or blood pressure measured using standard procedures, yielded similar results (appendix).

## Discussion

We used individual-level data from more than 1·7 million individuals in 48 independent cohort studies to compare the association of low socioeconomic status with mortality to those of six WHO 25 × 25 risk factor targets for the reduction of premature mortality. We found that the independent association between socioeconomic status and mortality is comparable in strength and consistency across countries to those for the 25 × 25 risk factors. Low socioeconomic status was associated with 2·1 YLLs between ages 40 and 85 years, while the corresponding years of life lost were 0·5 for high alcohol intake, 0·7 for obesity, 3·9 for diabetes, 1·6 for hypertension, 2·4 for physical inactivity and 4·8 for current smoking in men and women combined. These findings are largely consistent with previous studies,^17^, ^18^, ^19^ which used income or education as a measure of socioeconomic status.

The strong influence of socioeconomic factors on health, morbidity and mortality is well established,^3^, ^20^, ^21^, ^22^, ^23^, ^24^, ^25^ with studies showing a widening in inequalities in mortality^22^, ^25^ despite absolute inequalities falling in some countries.^22^, ^23^ Our study is one of the largest to examine the effect of low socioeconomic status on premature mortality and is to our knowledge the first large-scale study to directly compare the importance of socioeconomic circumstances as determinants of health with the six major risk factors targeted in global health strategies for the reduction of premature mortality. The association between low socioeconomic status and premature mortality was consistent across causes of death, whereas the 25 × 25 risk factors were generally more strongly associated with cardiovascular disease mortality than with cancer and with mortality of other causes.

We used occupational position as a proxy of socioeconomic status and social circumstances in general. This measure is one of the most commonly used indicators of socioeconomic status, data for this indicator were widely available across the cohort studies included in our analysis and occupational position is comparable between countries. Occupational position also has the advantage of reducing reverse causality—we assessed last known occupation, which is less likely to change with illness than is one's income. However, socioeconomic status is a complex factor that comprises several dimensions and by using a single indicator of socioeconomic status we might have underestimated its full effect on mortality. Addressing several components of socioeconomic status (ie, low occupational position, income poverty, low education) could be important for population health improvement.

This study has some important limitations. First, risk factors (ie, hypertension, physical activity, obesity, and diabetes) are interconnected making it difficult to establish their independent contribution. For example, low socioeconomic status might induce changes in one or more risk factors, but risk factors for chronic diseases might also reduce labour supply and earnings, thereby lowering socioeconomic status. Furthermore, factors other than those considered in the 25 × 25 list could be involved in the pathways between socioeconomic status and mortality. In view of these complex relationships, our estimates of the population attributable fraction, assuming unidirectional causal associations, should be interpreted with caution. Second, different measures of socioeconomic status can themselves be intertwined, and can influence risk factors for health or disease at different points over a person's life. For example, increased educational levels might contribute to increased life expectancy via multiple pathways including better occupational position, higher income, less smoking, reduced occupational hazard, more physical activity, healthier diet, increased self-care, and adherence to medical treatments.^26^ However, the finding that socioeconomic status is associated with death risk independently of conventional risk factors suggests that both socioeconomic adversity and 25 × 25 risk factors should be targeted by health strategies. Third, with broad two-level or three-level categorisations, the assessment of both socioeconomic status and risk factors was crude, potentially underestimating the strength of associations with mortality outcomes. However, the comparison between risk factors should be balanced because they were all measured with the same relative level of precision. The observed associations of smoking, physical activity, high alcohol intake, diabetes, and hypertension with mortality were comparable with those of previous studies.^27^, ^28^, ^29^, ^30^ The non-significant outcome observed between obesity and all-cause mortality in men might be an underestimate due to pre-existing morbidity leading to weight loss and increased mortality risk among lean or underweight individuals.^31^, ^32^ Heterogeneity in study-specific estimates was generally low for occupational position, but larger for some of the risk factors (appendix). This difference could be due to varying degrees of precision in the measurement of the 25 × 25 risk factors in the different cohorts, and random-effect meta-analysis partially takes this uncertainty into account for the estimation of pooled effects. Finally, the cohort studies participating in the LIFEPATH consortium were from high-income countries. Thus, our results might not be generalisable to other populations. Previous studies suggest that socioeconomic factors and the 25 × 25 risk factors are also strong predictors of premature mortality in low and middle income countries.^33^ Further research should assess socioeconomic status and 25 × 25 risk factors in predicting mortality in different economic settings.

Despite these limitations, our study has important implications. Our findings suggest that existing global strategies and actions defined in the 25 × 25 health plan and the Global Burden of Diseases surveillance programme potentially exclude a major determinant of health from the agenda. A lack of consideration of the interrelation between social circumstances and health is also evident in the Sustainable Development Goals (SDGs): SDG 3 focuses on health but it makes no mention of the role of social circumstances. Similarly, SDG 1 and 4 focus on the elimination of poverty and the achievement of universal primary education but they do not mention reducing health inequalities as an explicit goal. Similar to the risk factors targeted by existing global health strategies, socioeconomic circumstances are modifiable by policies at the local, national, and international levels,^26^, ^34^ through interventions such as promotion of early childhood development, poverty reduction, improvements to access to high-quality education, enacting of compulsory schooling laws, and creation of safe home, school, and work environments.^8^, ^9^ Over the past decade, socioeconomic factors have started making their way into international agencies and global reports, as evidenced in the report of the WHO Commission on the Social Determinants of Health (CSDH) in 2008^26^ and in the Rio Political Declaration on the Social Determinants of Health.^35^ Although these efforts have raised awareness of socioeconomic inequalities in health, global prevention strategies still appear to be centred on the treatment of proximal risk factors. Such approaches fail to address powerful upstream structural solutions such as investment in early education programmes for children (allowing parents to work while their children are cared for) and work incentive programmes (ie, earned income tax credit) that might be a cost-effective way to reduce inequalities in health.^10^, ^36^, ^37^, ^38^ By showing low socioeconomic status has a comparable health effect to that of major risk factors, the results of our study suggest that socioeconomic circumstances, in addition to the 25 × 25 factors, should be treated as a target for local and global health strategies, health risk surveillance, interventions, and policy.

**This online publication has been corrected. The corrected version first appeared at thelancet.com on February 27, 2017**
