# Department of Error

**DOI:** 10.1016/S0140-6736(17)30606-2

**Published:** 2017-03-25

*Stringhini S, Carmeli C, Jokela M, et al. Socioeconomic status and the 25 × 25 risk factors as determinants of premature mortality: a multicohort study and meta-analysis of 1·7 million men and women.* Lancet *2017; **389:** 1229–37*—the declaration of interests for this Article had stated “PV reports grants from GlaxoSmithKline.” Two authors have the initials PV, Peter Vollenweider, to whom the interest refers, and Paolo Vineis, a last author of the paper. This has been amended to “PVo reports grants from GlaxoSmithKline.” This correction has been made online as of Feb 27, 2017, and the printed Article is correct.

