# Department of Error

**DOI:** 10.1016/S0140-6736(17)30311-2

**Published:** 2017-03-25

*Stringhini S, Carmeli C, Jokela M, et al. Socioeconomic status and the 25 × 25 risk factors as determinants of premature mortality: a multicohort study and meta-analysis of 1·7 million men and women.* Lancet *2017; **389:** 1229–37*—In the supplementary appendix for this Article, some forest plots were omitted for all-cause mortality that have now been added. This correction has been made to the online version as of Feb 7, 2017, and the printed Article is correct.

