# Supplementary material for: Socioeconomic status and the 25 × 25 risk factors as determinants of premature mortality: a multicohort study and meta-analysis of 1·7 million men and women
Source: Lancet. 2017 Mar 25;389(10075):1229–37. doi: 10.1016/S0140-6736(16)32380-7 (PMC5368415; doi:10.1016/S0140-6736(16)32380-7)
Supplement: Supplementary appendix [file mmc1.pdf]

THE LANCET

Supplementary appendix

This appendix formed part of the original submission and has been peer reviewed. We post it as supplied by the authors. **This online publication has been corrected. The corrected version first appeared at thelancet.com on February 7, 2017**

Supplement to: Stringhini S,Cristian C,Jokela M, et al, for the LIFEPATH consortium. Socioeconomic status and the 25 × 25 risk factors as determinants of premature mortality: a multicohort study and meta-analysis of 1·7 million men and women. *Lancet* 2017; published online Jan 31. http://dx.doi.org/10.1016/S0140-6736(16)32380-7.

Web appendices

Contents

Page

1. Details on the study design and participants of the cohorts included in the analysis — 1
2. Study characteristics — 5
3. Quality assessment of included studies — 7
4. Details on measurements of the 25x25 risk factors — 9
5. Sensitivity analyses — 11

Appendix 1. Details on the study design and participants of the cohorts included in the analysis

COLAUS. The CoLaus (COhorte LAUSannoise) is an ongoing prospective study assessing the clinical, biological and genetic determinants of cardiovascular disease in the city of Lausanne, Switzerland. The initial survey was conducted between 2003 and 2006 and included 6733 participants aged between 35 and 75 years; the first follow-up survey was conducted 5.5 years afterwards and included 5064 participants. In each survey, data on socio-economic status, lifestyle, mental status and cardiovascular risk factors is collected by questionnaire or clinical examination. http://www.colaus.ch/en/cls_home/cls_pro_home.htm

GAZEL. The GAZEL study was established in 1989 among employees of the French national gas and electricity company, Electricité de France-Gaz de France (EDF-GDF). At baseline (1989), 20,625 employees (15,011 men and 5,614 women), aged 35–50, gave consent to participate. The study design consists of an annual questionnaire used to collect data on health, lifestyle, individual, familial, social and occupational factors, and life events. http://www.gazel.inserm.fr/en/

CONSTANCES. The CONSTANCES cohort was established in late 2012. It was designed as a randomly selected representative sample of French adults aged 18-69 years at inception; 200,000 subjects will be included over a five-year period. At enrolment the participants fill questionnaires collecting data on health, lifestyle, individual, familial, social and occupational factors, and life events and benefits from a comprehensive health examination. The follow-up includes a yearly self-administered questionnaire, a health examination every 5 years and an annual linkage to social and health national databases (Zins et al, 2015). http://www.constances.fr/index_EN.php

WHITEHALL-II. The Whitehall II study was established in 1985 to examine the socioeconomic gradient in health among 10,308 London-based civil servants (6,895 men and 3,413 women) aged 35–55. Baseline examination (phase 1) took place during 1985–1988, and involved a clinical examination and a self-administered questionnaire containing sections on demographic characteristics, health, lifestyle factors, work characteristics, social support, and life events. https://www.ucl.ac.uk/whitehallII

ELSA. The English Longitudinal Study of Ageing (ELSA) is a panel study of a representative cohort of men and women living in England aged ≥50 years. It was designed as a sister study to the Health and Retirement Study in the USA and is multidisciplinary in orientation, involving the collection of economic,

social, psychological, cognitive, health, biological and genetic data. The study commenced in 2002, and the sample has been followed up every 2 years. Data are collected using computer-assisted personal interviews and self-completion questionnaires, with additional nurse visits for the assessment of biomarkers every 4 years. The original sample consisted of 11 391 members ranging in age from 50 to 100 years. http://www.elsa-project.ac.uk/

EPIC. EPIC is a multicenter prospective cohort study, which recruited 518,408 volunteers from 23 centres in 10 countries (Sweden, Denmark, Norway, the Netherlands, United Kingdom, France, Germany, Spain, Italy, and Greece) between 1992 and 2000. In brief, the study population included volunteers aged mostly 25 to 70 years at the time of recruitment. Informed consent forms were filled at each local centre and the study was approved by the Institutional Review Board at the International Agency for Research on Cancer (IARC) and the local ethical committees. Lifestyle questionnaires included questions on dietary items, education, occupation, previous illness, alcohol and tobacco consumption, and physical activity. Diet was measured by extensive country-specific validated dietary questionnaires, aiming to provide high compliance rates and detect variations in dietary habits. Cancer incidence, mortality data and incident CVD events were obtained at the regional level. For Denmark, Italy, the Netherlands, Spain, Sweden, and the United Kingdom, such information was ascertained by means of record linkages with cancer registries, boards of health, and death indexes. Active follow-up by mail or telephone with participants, municipal registries, regional health departments, physicians and hospitals were adopted in Germany, Greece, and France. We are including in Lifepath centres from EPIC Italy (N=34,148) with the exclusion of Florence. http://epic.iarc.fr/centers/italy.php

E3N. The E3N study (Étude Epidémiologique auprès de femmes de la Mutuelle Générale de l'Education Nationale) is a prospective cohort of 98,995 women aged 40-65 years at recruitment in 1990 and it constitutes the French component of EPIC. The study was established to produce a large mass of data relevant for the identification of environmental and molecular causes of cancer and other chronic diseases, and to contribute to the development of effective public health strategies. Data on residence, education, early life events, exposures, lifestyle factors and life events were collected at baseline and follow-up questionnaires were sent every 2-3 years with more than 80% response. Mortality data were obtained by means of regular record linkage with various French mortality databases. http://www.e3n.fr/

MCCS. The Melbourne Collaborative Cohort Study is a prospective cohort study of 41,514 participants (24,469 women) living in Melbourne, Australia. Caucasian volunteers aged between 40 and 69 years were recruited in randomly selected census districts. At baseline (1990–1994), participants attended clinics where demographic, lifestyle and dietary information were collected and anthropometric measurements were performed. http://www.cancervic.org.au/research/epidemiology/health_2020/health2020-overview

EPIPORTO. The EPIPorto study was initiated in 1999 and recruited 2485 adult dwellers aged 18 years or more in the city of Porto, northwest of Portugal. Briefly, simple random digit dialing of landline telephones was used to select households. The vast majority of houses (>95%) had a landline telephone at the time of this procedure. We used a table of random numbers to define the last four digits that are specific to individual houses, assuming the local prefix codes to limit the universe to the city of Porto. Non-existing numbers, those corresponding to fax numbers or telephone numbers of non-individual subscribers were ignored. The household was considered unreachable after at least four dialing attempts at different hours and including week and weekend days. Within each household, a permanent resident aged 18 years or more was selected using simple random sampling. The proportion of participation was 70%. A follow-up evaluation was conducted from 2005 to 2008 (participation rate=68% of the baseline sample), by trained interviewers, using structured questionnaires and forms, following the same protocol for data collection as at baseline. In both evaluations, participants were invited to visit our Department at Medical School for an interview, which

included a questionnaire on social, demographic, behavioural and clinical data, and a physical examination including blood collection.

NCDS. The NCDS (National Child Development Study) recruited 17 415 babies born in one week of 1958 (98.2% of all births that week) in Great Britain (England, Scotland and Wales). Participants were surveyed at birth and ages 7, 11, 16, 23, 33, 42, 44/45, 46 and 50. Information was collected on economic, medical, developmental and social aspects of participants' lives. At age 46/47 (the cut-off age of this study), a sub-sample of participants (n = 11881, 77.9% of the target) took part in a biomedical survey (Power & Elliott, 2006). http://www.cls.ioe.ac.uk/page.aspx?&sitesectionid=724&sitesectiontitle=Welcome+to+the+1958+National+Child+Development+Study

WHIP. The Work History Panel is based on a sample of individual-level data from the Social Security Administration archives in Italy, covering almost 8% of all Italian workers employed in the private sector in 1985-2010. Unlike all other cohorts in LIFEPATH, it does not have biological samples, but it provides very rich information on income, pensions, unemployment benefits, disability indemnities, workplace and job contracts, linked to hospital and mortality follow-up. http://www.eui.eu/Research/Library/ResearchGuides/Economics/Statistics/DataPortal/WHIP.aspx

NHANES. The National Health and Nutrition Examination Survey (NHANES) is a program of studies designed to assess the health and nutritional status of adults and children in the United States. The survey is unique in that it combines interviews and physical examinations. NHANES is a major program of the National Center for Health Statistics (NCHS). NCHS is part of the Centers for Disease Control and Prevention (CDC) and has the responsibility for producing vital and health statistics for the Nation.The NHANES program began in the early 1960s and has been conducted as a series of surveys focusing on different population groups or health topics. In 1999, the survey became a continuous program that has a changing focus on a variety of health and nutrition measurements to meet emerging needs. The survey examines a nationally representative sample of about 5,000 persons each year. These persons are located in counties across the country, 15 of which are visited each year. The NHANES interview includes demographic, socioeconomic, dietary, and health-related questions. The examination component consists of medical, dental, and physiological measurements, as well as laboratory tests administered by highly trained medical personnel. http://www.cdc.gov/nchs/nhanes/

NHIS. The National Health Interview Survey (NHIS) is the principal source of information on the health of the civilian non-institutionalized population of the United States and is one of the major data collection programs of the National Center for Health Statistics (NCHS) which is part of the Centers for Disease Control and Prevention (CDC). The National Health Survey Act of 1956 provided for a continuing survey and special studies to secure accurate and current statistical information on the amount, distribution, and effects of illness and disability in the United States and the services rendered for or because of such conditions. The survey referred to in the Act, now called the National Health Interview Survey, was initiated in July 1957. While the NHIS has been conducted continuously since 1957, the content of the survey has been updated about every 10-15 years. In 1996, a substantially revised NHIS questionnaire began field testing. http://www.cdc.gov/nchs/nhis/

Alameda County Study. The Alameda County Study was designed to investigate normal daily routines and social-support factors to determine which might be risk factors for poor health and mortality in a real community.In 1965, a probability sample of the population of Alameda County, California was invited to participate in a study on health status, social networks, and other personal characteristics. The follow-up group contained 6,928 participants who completed questionnaires and were followed at intervals for up to 20 years after the initial investigation.http://www.epi.umn.edu/cvdepi/study-synopsis/alameda-county-study/

HALS. Health and Lifestyle Survey was undertaken in County Durham and Darlington (United Kingdom) in order to inform health strategies of the Primary Care Trusts. A stratified random sample of the population was asked to participate and a questionnaire was posted to those people who consented. The compelling message this survey sends out is the importance of deprivation in health inequalities and behavioural risks. Deprivation is a complex interaction of factors, which include environment, income, education, housing, employment and social capacity. Therefore to improve health and reduce inequalities, the NHS cannot act in isolation. Without co-ordinated action at all levels and across all agencies, no improvement will be seen in this unacceptable variation. https://www.nepho.org.uk/publications/487/Health_and_Lifestyle_Survey

HRS. The University of Michigan Health and Retirement Study (HRS) is a longitudinal panel study that surveys a representative sample of approximately 20,000 people in America over the age of 50 every two years. Supported by the National Institute on Aging (NIA U01AG009740) and the Social Security Administration, the HRS explores the changes in labor force participation and the health transitions that individuals undergo toward the end of their work lives and in the years that follow. Since its launch in 1992, the study has collected information about income, work, assets, pension plans, health insurance, disability, physical health and functioning, cognitive functioning, and health care expenditures. Through its unique and in-depth interviews, the HRS provides an invaluable and growing body of multidisciplinary data that researchers can use to address important questions about the challenges and opportunities of aging. http://hrsonline.isr.umich.edu/index.php?p=start

MIDUS. The first national survey of Midlife Development in the U.S. (MIDUS) was conducted in 1995/96 by the MacArthur Foundation Research Network on Successful Midlife Development. The study was conceived by a multidisciplinary team of scholars from fields of psychology, sociology, epidemiology, demography, anthropology, medicine, and health care policy. Their collective aim was to investigate the role of behavioral, psychological, and social factors in accounting for age-related variations in health and well-being in a national sample of Americans. In addition to a national probability sample (N = 3,487), the study included over-samples in select metropolitan areas (N = 757), a sample of siblings (N = 950) of the main respondents, and a national sample of twin pairs (N=1,914). http://midus.wisc.edu/scopeofstudy.php

WLSG and **WLSS**. The Wisconsin Longitudinal Study has followed a random sample of 10317 participants (5326 women, 4991 men) who were born between 1937 and 1940 and who graduated from Wisconsin high schools in 1957 (graduate sample, WLSG). After baseline data collection in 1957, survey data have been collected from the participants or their parents in 1964, 1975, 1992/3, and 2003/5. The present study used data from the 1993/1994 follow-up. The WLS sample is broadly representative of white, non-Hispanic American men and women who have completed at least a high school education (among Americans aged 50 to 54 in 1990 and 1991, approximately 66 percent were non-Hispanic white persons who completed at least 12 years of schooling). It is estimated that about 75 percent of Wisconsin youth graduated from high school in the late 1950s – everyone in the primary WLS sample graduated from high school. In addition to the main sample of the 1957 high school graduates, the WLS has also collected data on a selected sibling of a sample of the graduates (sibling sample, WLSS). The data collection in adulthood has been very similar although not entirely identical for the siblings as for the graduates. For the present purposes, the sibling sample was analyzed separately from the graduate sample, because the sampling frame of the individuals for the graduate cohort and sibling cohort was considered to sufficiently to justify the decision of not combining the samples. Baseline data were from the 1994/1995 follow-up.

Appendix 2. Study characteristics

Study	Baseline Year	Country	N participants<br>Men / Women	Mean follow-up<br>Men / Women	Age at baseline, mean (SD)<br>Men / Women	Origin
COLAUS	2003—2006	Switzerland, Lausanne	3189<br>3544	6·2<br>6·2	52·2 (10·7)<br>53·0 (10·7)	Population-based
EPIPORTO	1995–2005	Portugal, Porto	946<br>1539	6·8<br>6·4	53·4 (15·8)<br>52·6 (15·2)	Population-based
GAZEL	1989	France	15011<br>5614	25·3<br>25·8	44·5 (2·9)<br>41·7 (4·2)	Occupational cohort (EDF-GDF employees)
EPIC Italy	1992–1998	Italy, 4 cities	11657<br>22494	16·0<br>15·4	49·6 (7·6)<br>49·6 (8·2)	Population based
ELSA	2004–2005	United Kingdom, England	3948<br>4831	7·3<br>7·5	66·1 (9·5)<br>66·9 (10·0)	Population based
NCDS	1999–2005	United Kingdom	5882<br>5998	7·5<br>7·7	45·0 (2·9)<br>45·2 (2·1)	Birth cohort/population based
WHITEHALL II	1991–1994	United Kingdom, London	6057<br>2758	20·3<br>20·3	49·9 (6·0)<br>51·1 (6·2)	Occupational cohort (British civil servants)
MCCS	1990–1994	Australia	17045<br>24469	17·0<br>17·8	55·8 (8·8)<br>55·0 (8·6)	Population based
E3N	1989–1991	France	–<br>98995	–<br>16·2	–<br>49·4 (6·7)	Occupational cohort (employees of the education sector)
WHIP	1990–2012	Italy	146279<br>77304	11·8<br>11·1	57·8 (4·0)<br>57·6 (3·2)	Occupational cohort (random sample of workers retired from private enterprises
NHANES I	1971-1974	USA	5802<br>8588	16·7<br>19·0	51·4 (15·3)<br>47·1 (15·5)	Population-based
NHANES II	1976-1980	USA	4336<br>4885	12·8<br>13·7	54·3 (13·3)<br>54·3 (13·4)	Population-based
NHANES III	1988-1994	USA	8461<br>9638	13·0<br>13·4	47·1 (20.5)<br>46.6 (20.5)	Population-based
NHANES 1999	1999-2000	USA	2300<br>2575	10·5<br>10·9	47.9 (20.9)<br>46.3 (20.8)	Population-based
NHANES 2001	2001-2002	USA	2835<br>3141	9·1<br>9·4	47·2 (20·5)<br>46·6 (21·0)	Population-based
NHANES 2003	2003-2004	USA	2688<br>2916	7·4<br>7·6	47·6 (21·0)<br>47·4 (21·2)	Population-based
NHANES 2005	2005-2006	USA	2669<br>2882	5·7<br>5·8	46.2 (20·2)<br>44.2 (20·3)	Population-based
NHANES 2007	2007-2008	USA	2903<br>3017	3·9<br>3·9	50·6 (17·9)<br>51·0 (18·0)	Population-based
NHIS 1986	1986	USA	20026<br>22568	21·8<br>22·5	42·8 (17·3)<br>44·2 (18·1)	Population-based
NHIS 1987	1987	USA	39293<br>44934	21·2<br>21·8	43·1 (17·2)<br>44·4 (18·1)	Population-based
NHIS 1988	1988	USA	39505<br>44880	20·5<br>21·1	43·4 (17·2)<br>44·8 (18·1)	Population-based
NHIS 1989	1989	USA	37790<br>42895	19·9<br>20·3	43·3 (17·1)<br>44·7 (18·0)	Population-based
NHIS 1990	1990	USA	38710<br>44009	19·2<br>19·6	43·3 (17·1)<br>44·7 (18·0)	Population-based

NHIS 1991	1991	USA	38656<br>43746	18·4<br>18·9	43·7 (17·0)<br>44·9 (17·9)	Population-based
NHIS 1992	1992	USA	41026<br>46230	18·5<br>18·9	43·4 (16·9)<br>44·9 (17·9)	Population-based
NHIS 1993	1993	USA	35413<br>39840	17·0<br>17·3	43·7 (16·9)<br>45·1 (17·8)	Population-based
NHIS 1994	1994	USA	37081<br>41877	16·1<br>16·4	44·1 (16·9)<br>45·6 (17·9)	Population-based
NHIS 1995	1995	USA	32310<br>36326	15·4<br>15·7	43·7 (16·8)<br>44·9 (17·7)	Population-based
NHIS 1996	1996	USA	20068<br>22270	14·7<br>14·8	43·5 (16·6)<br>44·8 (17·5)	Population-based
NHIS 1997	1997	USA	14797<br>19582	13·8<br>14·0	45·2 (17·0)<br>46·8 (18·5)	Population-based
NHIS 1998	1998	USA	13448<br>17129	13·0<br>13·1	45·5 (17·2)<br>47·3 (18·3)	Population-based
NHIS 1999	1999	USA	12503<br>16572	12·2<br>12·3	45·9 (17·4)<br>47·4 (18·3)	Population-based
NHIS 2000	2000	USA	13230<br>17363	11·4<br>11·5	45·4 (17·2)<br>47·1 (18·4)	Population-based
NHIS 2001	2001	USA	13663<br>17692	10·5<br>10·6	45·3 (17·1)<br>47·1 (18·2)	Population-based
NHIS 2002	2002	USA	12275<br>15934	9·6<br>9·7	45·7 (17·2)<br>47·4 (18·5)	Population-based
NHIS 2003	2003	USA	12275<br>15934	8·8<br>8·8	45·7 (17·2)<br>47·6 (18·4)	Population-based
NHIS 2004	2004	USA	12977<br>16215	8·2<br>8·2	46·2 (17·2)<br>48·0 (18·5)	Population-based
NHIS 2005	2005	USA	12791<br>16338	6·9<br>7·0	46·8 (17·3)<br>48·1 (18·3)	Population-based
NHIS 2006	2006	USA	10355<br>13063	6·0<br>6·1	46·1 (17.4)<br>47·7 (18·5)	Population-based
NHIS 2007	2007	USA	10056<br>12636	5·1<br>5·1	46·7 (17·4)<br>48·0 (18·3)	Population-based
NHIS 2008	2008	USA	9345<br>12017	4·2<br>4·2	46·9 (17·4)<br>48·7 (18·5)	Population-based
NHIS 2009	2009	USA	12105<br>15279	3·2<br>3·2	47·0 (17·6)<br>48·3 (18·3)	Population-based
Alameda County	1965	USA, California	3157<br>3769	27·5<br>28·9	43·6 (15·5)<br>43·9 (16·6)	Population-based
HALS	1984-85	United Kingdom	3903<br>5098	20·3<br>21·3	45·6 (17·6)<br>46·1 (17·8)	Population-based
HRS	1992	USA	5866<br>6783	16·3<br>17·9	57·0 (5·4)<br>53·7 (5·5)	Population-based
MIDUS	1995-96	USA	3001<br>3319	11·4<br>11·5	46·8 (12·8)<br>47·0 (13·0)	Occupational cohort, midwives
WLSG	1992-93	USA, Wisconsin	3971<br>4473	15·0<br>15·2	54·2 (0·5)<br>54·1 (0·5)	High school graduates
WLSS	1994-95	USA, Wisconsin	2563<br>2756	12·8<br>13·0	52·7 (7·4)<br>53·0 (7·3)	Siblings of high school graduates

CI: Confidence Interval. SD: Standard Deviation.ELSA: English Longitudinal Study on Ageing; NCDS: National Child Development Study; MCCS: Melbourne Collaborative Cohort Study; NHANES: National Health and Nutrition Examination Survey; NHIS: National Health Interview Survey; HALS: Health and Lifestyle Survey; HRS: Health and Retirement Study; MIDUS: Midwives in the United States Study; WLSG: Wisconsin Longitudinal Study (Graduate sample); WLSS: Wisconsin Longitudinal Study (Siblings sample).

Appendix 3. Quality assessment of included studies

To assess study quality, we evaluated potential bias in the following domains:

1. Was selection of exposed and non-exposed cohorts drawn from the same population?
2. Can we be confident in the assessment of exposure?
3. Can we be confident that the outcome of interest was not present at start of the study?
4. Did the statistical analysis adjust for the confounding variables?
5. Can we be confident in the assessment of the presence or absence of confounding factors?
6. Can we be confident in the assessment of outcome?
7. Was the follow up of cohorts adequate?

The studies were evaluated in relation to each question using 4 categories: ++ "definitely yes"," + probably/mostly yes", - "probably/mostly no", and --"definitely no". The quality of the study was considered high if all domains were evaluated favourably (++ or +).

	1 Exposed and unexposed from the same population	2 Confidence in exposure assessment	3 Confidence in exclusion of prevalent cases	4 Comprehensive adjustments	5 Confidence in confounders assessment	6 Confidence in outcome assessment	7 Adequate follow-up	HIGH QUALITY
EPIPORTO	++	++	++	++	++	++	+	Yes
ELSA	++	++	++	++	+	++	+	Yes
Whitehall II	++	++	++	++	++	++	++	Yes
NHANES I	++	++	++	++	++	++	++	Yes
NHANES II	++	++	++	++	++	++	++	Yes
NHANES III	++	++	++	++	++	++	++	Yes
NHANES 1999	++	++	++	++	++	++	++	Yes
NHANES 2001	++	++	++	++	++	++	+	Yes
NHANES 2003	++	++	++	++	++	++	+	Yes
NHANES 2005	++	++	++	++	++	++	+	Yes
NHANES 2007	++	++	++	++	++	++	+	Yes
NHIS 1997	++	++	++	++	+	++	++	Yes
NHIS 1998	++	++	++	++	+	++	++	Yes
NHIS 1999	++	++	++	++	+	++	++	Yes
NHIS 2000	++	++	++	++	+	++	++	Yes
NHIS 2001	++	++	++	++	+	++	++	Yes
NHIS 2002	++	++	++	++	+	++	+	Yes
NHIS 2003	++	++	++	++	+	++	+	Yes
NHIS 2004	++	++	++	++	+	++	+	Yes
NHIS 2005	++	++	++	++	+	++	+	Yes
NHIS 2006	++	++	++	++	+	++	+	Yes
NHIS 2007	++	++	++	++	+	++	+	Yes
NHIS 2008	++	++	++	++	+	++	+	Yes
NHIS 2009	++	++	++	++	+	++	+	Yes
Alameda County	++	++	++	++	+	++	++	Yes
HALS	++	++	++	++	++	++	++	Yes
HRS	++	++	++	++	+	++	++	Yes
MIDUS	++	++	++	++	+	++	++	Yes
WLSG	++	++	++	++	+	++	++	Yes
WLSS	++	++	++	++	+	++	++	Yes
COLAUS	-	++	++	++	++	++	+	No
GAZEL	-	++	++	++	+	++	++	No
EPIC Italy	-	++	++	++	++	++	++	No
NCDS	++	++	++	-	++	++	+	No

MCCS	-	-	+ +	+ +	+ +	+ +	+ +	No
E3N	-	+ +	+ +	-	+	+ +	+ +	No
WHIP	+ +	+ +	+ +	-	-	+ +	+ +	No
NHIS 1986	+ +	+ +	+ +	-	+	+ +	+ +	No
NHIS 1987	+ +	+ +	+ +	-	+	+ +	+ +	No
NHIS 1988	+ +	+ +	+ +	-	+	+ +	+ +	No
NHIS 1989	+ +	+ +	+ +	-	+	+ +	+ +	No
NHIS 1990	+ +	+ +	+ +	-	+	+ +	+ +	No
NHIS 1991	+ +	+ +	+ +	-	+	+ +	+ +	No
NHIS 1992	+ +	+ +	+ +	-	+	+ +	+ +	No
NHIS 1993	+ +	+ +	+ +	-	+	+ +	+ +	No
NHIS 1994	+ +	+ +	+ +	-	+	+ +	+ +	No
NHIS 1995	+ +	+ +	+ +	-	+	+ +	+ +	No
NHIS 1996	+ +	+ +	+ +	-	+	+ +	+ +	No

++ = *definitely yes*; + = *probably/mostly yes*; - = *probably/mostly no*; - - = *definitely no.*

In all studies, individuals with high and low SES were from the same cohort and response at baseline was 60% or higher in all studies, except COLAUS, GAZEL and E3N for which it was less than 50%. In MCCS and EPIC Italy, the eligible population was not clearly defined as the participants were recruited from universities, cafeterias etc. Self-reported assessment was considered reliable for SES, but this was missing for MCCS. There were no exclusions to prevalent cases in mortality analyses. The 25 x 25 risk factors (smoking, blood pressure, body mass index, diabetes, alcohol consumption, physical activity) were assessed in all studies except NCDS, E3N, WHIP and NHIS 1986-1996 which missed assessment of one or more 25x25 risk factors. Clinic measurements were used in COLAUS, EPIPORTO, EPIC ITALY, MCCS, NCDS, Whitehall II, NHANES I-III, 1999, 2001, 2003, 2005 and 2007, whereas other studies measured baseline variables using self-report assessment. Mortality ascertainment was reliable in all studies. The follow-up for mortality was optimal (>10 years) in most studies and adequate (>3 years) in all studies. Of the 48 studies, 30 were of high quality with all 7 domains evaluated favorably.

Appendix 4. Details on measurements of the 25x25 risk factors

Measures. *Socioeconomic position (SES)* was measured through occupational position, obtained from the European Socio-economic Classification (ESEC)(https://www.iser.essex.ac.uk/archives/esec/user-guide), which categorizes occupational position in 9 classes. For the LIFEAPTH Consortium harmonisation, a variable in 3 classes was obtained: High, including higher professionals and managers, lower professionals and managers, higher clerical, services and sales workers (Class 1, 2 and 3 ESEC); Intermediate, including small employers and self-employed, farmers, lower supervisors and technicians (Class 4, 5, and 6 ESEC); and low, including lower clerical, services and sales workers, skilled workers, semi- and unskilled workers (Class 7, 8, 9 ESEC). Occupational position was the last known one (current occupation for working individuals and last know occupation for those not currently working). For E3N, occupational position was current occupation 2-yr after baseline. In the cohorts from the United States, a similar occupational categorization was applied based on occupational groups defined by the Standard Occupational Classification codes.

Smoking was self-reported and was categorized into current smoking for individuals currently smoking, former smoking for individuals not currently smoking but who smoke in the past, and never smoking for individuals who never smoke.

Alcohol consumption was measured in alcohol units. For most cohorts, it was the average number of alcohol units over a week. For ELSA, it was the highest intake over a week. For EPIC-Italy, EPIPORTO and MCCS alcohol intake was measured in grams and they were converted to alcohol units by assuming that 12grams equal one alcohol unit. For E3N and GAZEL, alcohol consumption was available 3-yr after baseline.

High alcohol intake was defined as a consumption exceeding 21 alcohol units for men and 14 for women. Abstinence was defined as no alcohol consumption, and moderate intake was defined as consumption less or equal 21 alcohol units for men and 14 for women.

Physical inactivity was considered as less than 20 minutes of physical activity per week in COLAUS, neither moderate nor intense physical activity per day in EPIPORTO, neither moderate nor intense physical activity per week in E3N, none/light activity only for ELSA, as less than 1 hour of moderate and vigorous physical activity per week in WHITEHALL II, no physical activity in EPIC Italy, as not practicing sport in GAZEL, as a physical activity score of zero in MCCS. Physical inactivity was determined as participating in no weekly leisure-time aerobic activity that lasted at least 10 minutes in the NHIS; no moderate or vigorous physical activity over the past 30 days in the NHANES; no moderate or vigorous physical activity at least once a week in the HRS, MIDUS, WLSG, and WLSS; as not participating in sports or exercise in the HALS and ACS.

Body mass index (BMI) was measured as weight (Kg) divided by the square of height (m^2). *Obesity* is defined as BMI$\geq$30; *Overweight* is defined as BMI$\geq$25 and BMI<30; *Normal BMI* is defined as BMI$\geq$18·5 and BMI<25. For NCDS, the self-reported values of weight and height at wave 6 were used whenever the measured values during the biomedical survey at 2003 were not available. For E3N and GAZEL the values were self-reported. Height and weight were measured in the NHANES and HALS, and self-reported in ACS, HRS, NHIS, MIDUS, WLSG, and WLSS.

Hypertension was defined as the presence of at least one of the following conditions: systolic blood pressure$\geq$140 mmHg, recorded diastolic blood pressure$\geq$90 mmHg, current intake of anti-hypertensive medication, self-report. For most cohorts, the four conditions were available, for GAZEL only the self-reported information was available and for E3N only self-reported blood pressure values, and for NCDS knowledge of ant-hypertensive medication was not available. For most cohorts blood pressure values were the average of two readings, while EPIC-Italy had only one. Blood pressure was measured as the mean of two or three readings in the NHANES and HALS, and self-reported in ACS, HRS, MIDUS, WLSG, and WLSS.

Diabetes was defined as the presence of at least one of the following conditions: fasting glucose≥7 mmol/L, 2h postload glucose≥11·1 mmol/L, glycated hemoglobin ≥6·5%, self-report. All but E3N have self-reported information, WHITEHALL-II further has both fasting glucose and 2h post load glucose, ELSA and NCDS have glycated hemoglobin, and EPIPORTO and COLAUS fasting glucose. In the NHANES, diabetes was determined based on fasting glucose (except for NHANES I in which diabetes status was self-reported) or taking medication, and self-reported as whether the participant had been diagnosed of diabetes by a medical doctor in the ACS, HALS, HRS, MIDUS, NHIS, WLSG, and WLSS

Marital status was categorized as married/cohabiting individuals and single/separated/divorced/widowed individuals.

Race/ethnicity was categorized as white and non white individuals. Cohorts EPIC, GAZEL, E3N, EPIPORTO, MCCS only included individuals of white race/ethnicity. In the US cohorts, race/ethnicity was categorized with 4 groups (non-Hispanic white, black, Hispanic, other).

Appendix 5. Sensitivity analyses

ANALYSES RESTRICTED TO EUROPEAN COHORTS - MEN

Risk factor	N Cohorts	N Deaths	N Participants	HR [95% CI]	I^2/tau^2
Low SES (Ref. High)	9	26976	175314	1·52 [1·44;1.60]	0%/0
Current Smoking (Ref. Never	8	5957	47352	2·36 [2·06;2·70]	52·1%/0·0130
Diabetes	8	5947	46027	1·83 [1·37;2·44]	71·7%/0·0613
Physical Inactivity	7	5365	39682	1·46 [1·22;1·76]	82·5%/0·0347
High Alcohol Intake (Ref. Moderate)	8	5444	44159	1·31 [1·11;1·56]	74·1%/0·0297
Hypertension	8	6052	47982	1·20 [1·02;1·42]	81·4%/0·0351
Obesity (Ref. Normal BMI)	8	5059	44045	1·30 [1·00;1·69]	82·5%/0·0789

CI: Confidence Interval; HR: Hazard Ratio; SES: SocioEconomic Status; I^2: heterogeneity statistic; tau^2: inter-cohort variance

ANALYSES RESTRICTED TO EUROPEAN COHORTS - WOMEN

Risk factor	N Cohorts	N Deaths	N Participants	HR [95% CI]	I^2/tau^2
Low SES (Ref. High)	10	12707	154422	1·29 [1·15;1·44]	30·1%/0·0065
Current Smoking (Ref. Never)	9	12739	144473	1·82 [1·59;2·07]	57·7%/0·0101
Diabetes	8	4250	48883	1·76 [1·41;2·19]	48·9%/0·0372
Physical Inactivity	8	10771	118113	1·41 [1·18;1·68]	80·7%/0·0239
High Alcohol Intake (Ref. Moderate)	9	9813	118430	1·24 [1·08;1·42]	28·3%/0·0072
Hypertension	9	9286	105233	1·13 [1·02;1·26]	61·6%/0·0110
Obesity (Ref. Normal BMI)	9	11484	136259	1·44 [1·18;1·74]	65·1%/0·0232

CI: Confidence Interval; HR: Hazard Ratio; SES: SocioEconomic Status; I^2: heterogeneity statistic; tau^2: inter-cohort variance

ANALYSES RESTRICTED TO PREMATURE MORTALITY (<70 years) - MEN

Risk factor	N Cohorts	N Deaths	N Participants	HR [95% CI]	I^2/tau^2
Low SES (Ref. High)	46	66809	591735	1·61 [1·55;1·67]	24·4%/0·0030
Current Smoking (Ref. Never)	35	21443	242229	2·63 [2·52;2·75]	0%/0
Diabetes	35	27087	239766	2·27 [2·08;2·48]	48·6%/0·0240
Physical Inactivity	34	21567	233758	1·65 [1·54; 1·77]	65·3%/0·0241
High Alcohol Intake (Ref. Moderate)	35	18736	216334	1·71 [1·53;1·92]	62·9%/0·0494
Hypertension	35	22219	245666	1·57 [1·46;1·70]	68·9%/0·0291
Obesity (Ref. Normal BMI)	46	86321	580697	1·23 [1·16;1·32]	69·3%/0·0189

CI: Confidence Interval; HR: Hazard Ratio; SES:SocioEconomic Status; I^2: heterogeneity statistic; tau^2: inter-cohort variance

ANALYSES RESTRICTED TO PREMATURE MORTALITY (<70 years) - WOMEN

Risk factor	N Cohorts	N Deaths	N Participants	HR [95% CI]	I^2/tau^2
Low SES (Ref. High)	47	38140	543745	1·51 [1·41;1·63]	39·8%/0·0155

Current Smoking (Ref. Never)	36	20734	382867	2·30 [2·13;2·49]	72·1%/0·0407
Diabetes	35	21247	287861	2·73 [2·48;3·01]	51·2%/0·0340
Physical Inactivity	35	20386	358133	1·67 [1·53; 1·83]	71·9%/0·0354
High Alcohol Intake (Ref. Moderate)	35	17201	320077	1·86 [1·60;2·16]	61·3%/0·0871
Hypertension	36	19463	348088	1·56 [1·44;1·68]	79·9%/0·0599
Obesity (Ref. Normal BMI)	47	73729	730732	1·53 [1·44;1·62]	61·6%/0·0156

CI: Confidence Interval; HR: Hazard Ratio; SES: SocioEconomic Status; I^2: heterogeneity statistic; tau^2: inter-cohort variance

ANALYSES RESTRICTED TO HIGH QUALITY STUDIES – MEN (see Appendix 3 for definition)

Risk factor	N Cohorts	N Deaths	N Participants	HR [95% CI]	I^2/tau^2
Low SES (Ref. High)	30	16647	150760	1.43 [1.37;1.5]	10.83%/0.0014
Current Smoking (Ref. Never)	30	32294	215016	1.66 [1.53;1.8]	79.02%/0.0265
Diabetes	30	33563	223439	1.3 [1.24;1.36]	62.58%/0.0073
Physical Inactivity	30	31667	215696	0.95 [0.87;1.04]	77.76%/0.0271
High Alcohol Intake (Ref. Moderate)	30	32984	217822	1.64 [1.54;1.73]	75.45%/0.0135
Hypertension	30	26297	189377	1.55 [1.41;1.7]	63.22%/0.0318
Obesity (Ref. Normal BMI)	30	32074	218273	2.14 [2.02;2.26]	66.97%/0.0152

CI: Confidence Interval; HR: Hazard Ratio; SES: SocioEconomic Status; I^2: heterogeneity statistic; tau^2: inter-cohort variance

ANALYSES RESTRICTED TO HIGH QUALITY STUDIES – WOMEN (see Appendix 3 for definition)

Risk factor	N Cohorts	N Deaths	N Participants	HR [95% CI]	I^2/tau^2
Low SES (Ref. High)	30	11469	159845	1.38 [1.28;1.49]	33.78%/0.0122
Current Smoking (Ref. Never)	30	32611	267332	1.85 [1.69;2.02]	82.57%/0.032
Diabetes	30	33747	277091	1.3 [1.22;1.39]	76.25%/0.0147
Physical Inactivity	30	30116	255990	1.06 [0.99;1.15]	71.92%/0.0175
High Alcohol Intake (Ref. Moderate)	30	33243	271462	1.63 [1.54;1.72]	74.66%/0.0142
Hypertension	30	26738	235862	1.81 [1.56;2.1]	64.44%/0.1037
Obesity (Ref. Normal BMI)	30	32362	269815	2.05 [1.93;2.18]	71.89%/0.0173

CI: Confidence Interval; HR: Hazard Ratio; SES: SocioEconomic Status; I^2: heterogeneity statistic; tau^2: inter-cohort variance

ANALYSES OF THE ASSOCIATION BETWEEN SES AND ALL-CAUSE MORTALITY RESTRICTED TO PARTICIPANTS WITHOUT RISK FACTORS

Sub-groups	N Cohorts	N Deaths	N Participants	HR [95% CI]	I^2/tau^2
No Current Smoking	24	19656	297612	1·36 [1·29;1·44]	20·2%/0·0032
No Diabetes	23	17657	283544	1·53 [1·46;1·61]	3·2%/0·0004
No Hypertension	24	13889	261892	1·56 [1·47;1·65]	12·7%/0·0024
No Obesity	35	72393	747161	1·42 [1·39;1·46]	8·7%/0·0004
No Physical Inactivity	22	13728	212585	1·48 [1·38;1·60]	40·8%/0·0117
No Heavy Alcohol Intake	24	19257	302210	1·47 [1·39;1·54]	24·5%/0·0039
No Risks Factors	21	2223	68770	1·26 [1·11;1·42]	0·0%/0·0000

CI: Confidence Interval; HR: Hazard Ratio; SES:SocioEconomic Status; I^2: heterogeneity statistic; tau^2: inter-cohort variance

ANALYSES RESTRICTED TO COHORTS WITH MEASURED HEIGHT AND WEIGHT (N COHORTS=16)

Risk factor	N Deaths	N Participants	HR [95% CI]	I^2/tau^2
Obesity (Ref. Normal BMI) - Men	15333	77849	1·02 [0·87;1·20]	86·1%/0·0603
Obesity (Ref. Normal BMI) - Women	13089	101028	1·14 [1·01;1·29]	68·4%/0·0195

CI: Confidence Interval; HR: Hazard Ratio; I^2: heterogeneity statistic; tau^2: inter-cohort variance

ANALYSES RESTRICTED TO COHORTS WITH MEAN MORTALITY FOLLOW-UP >10 YEARS

Risk factor	N Deaths	N Participants	HR [95% CI]	I^2/tau^2
Obesity (Ref. Normal BMI) - Men, n=30	123213	523984	1·12 [1·05;1·21]	85·7%/0·0130
Obesity (Ref. Normal BMI) - Women, n=31	128248	681084	1·25 [1·18;1·31]	82·7%/0·0088

CI: Confidence Interval; HR: Hazard Ratio; I^2: heterogeneity statistic; tau^2: inter-cohort variance

ANALYSES RESTRICTED TO COHORTS WITH BLOOD PRESSURE MEASURED USING STANDARD PROCEDURES (N COHORTS=15)

Risk factor	N Deaths	N Participants	HR [95% CI]	I^2/tau^2
Hypertension - Men	16539	81323	1·19 [1·12;1·27]	50·4%/0·0056
Hypertension - Women	14592	106463	1·21 [1·12;1·31]	55·1%/0·0087

CI: Confidence Interval; HR: Hazard Ratio; I^2: heterogeneity statistic; tau^2: inter-cohort variance

Appendix 6. Forest plot of the study-specific age-, sex-, marital status- and race/ethnicity-adjusted hazard ratios for 25x25 risk factors and mortality

PLEASE SEE PDF

Age-, marital status- and race/ethnicity-adjusted hazard ratios for all-cause mortality in current smokers compared to never smokers – MEN

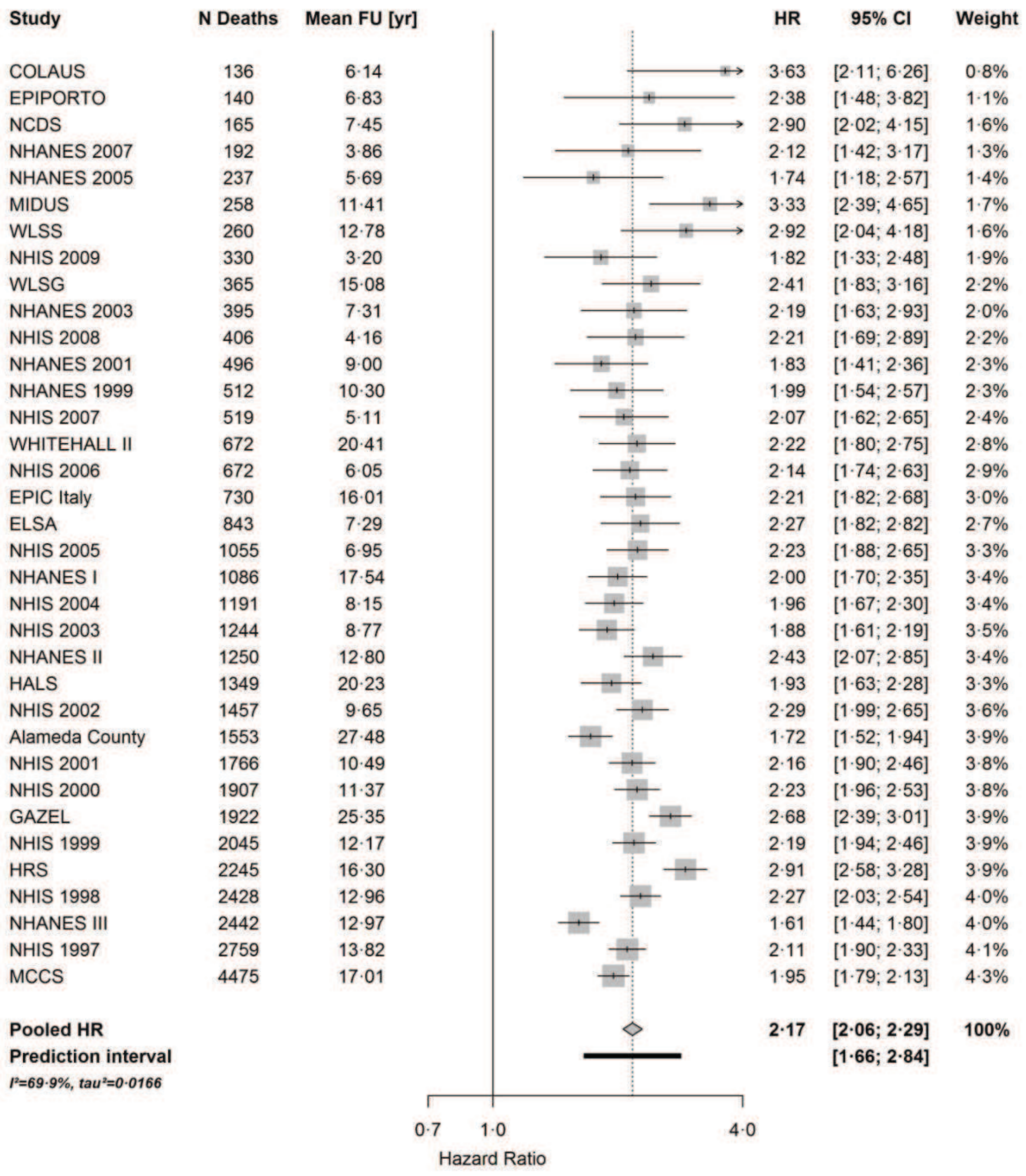

Pooled HR is represented with a gray diamond and the 95% prediction interval with a black bar. I^2 statistic is the percentage of between study heterogeneity; tau^2 statistics measures the inter-study variance. The prediction interval provides a predicted range for the true association between smoking and all-cause mortality.

Age-, marital status- and race/ethnicity-adjusted hazard ratios for all-cause mortality in current smokers compared to never smokers – WOMEN

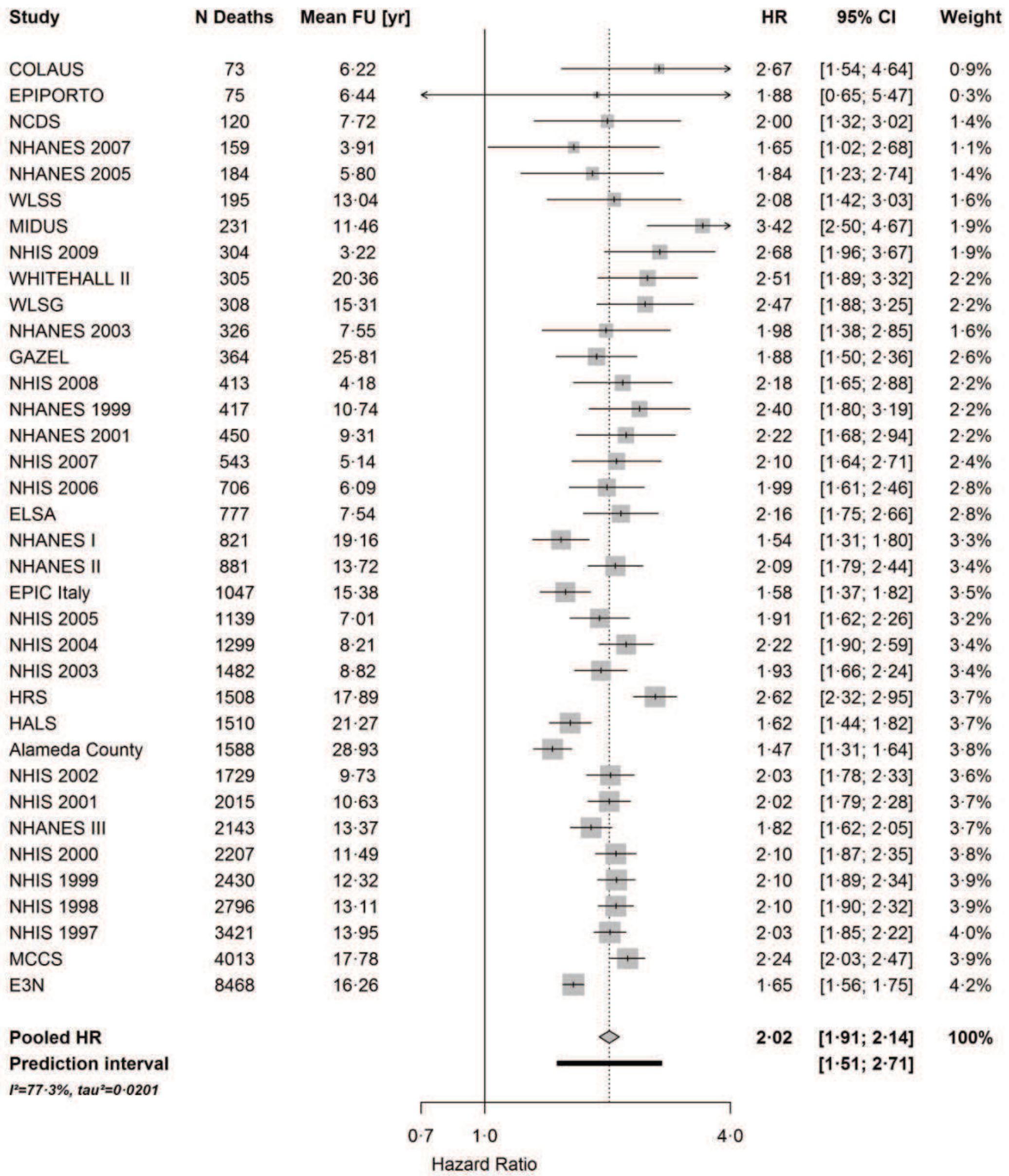

Pooled HR is represented with a gray diamond and the 95% prediction interval with a black bar. I^2 statistic is the percentage of between study heterogeneity; tau^2 statistics measures the inter-study variance. The prediction interval provides a predicted range for the true association between smoking and all-cause mortality.

Age-, marital status- and race/ethnicity-adjusted hazard ratios for all-cause mortality in diabetics compared to non diabetics – MEN

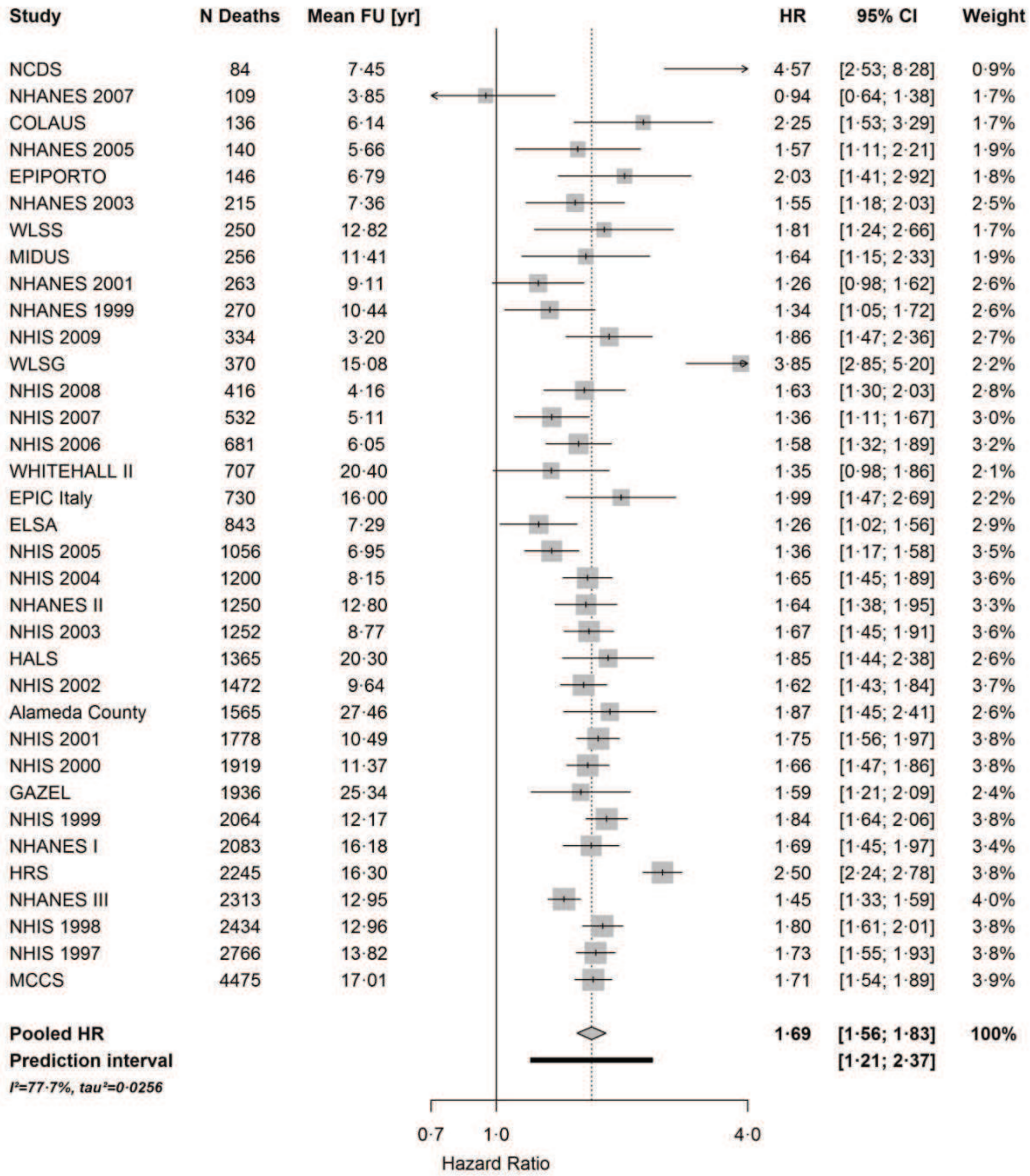

Pooled HR is represented with a gray diamond and the 95% prediction interval with a black bar. I^2 statistic is the percentage of between study heterogeneity; tau^2 statistics measures the inter-study variance. The prediction interval provides a predicted range for the true association between diabetes and all-cause mortality.

Age-, marital status- and race/ethnicity-adjusted hazard ratios for all-cause mortality in diabetics compared to non diabetics – WOMEN

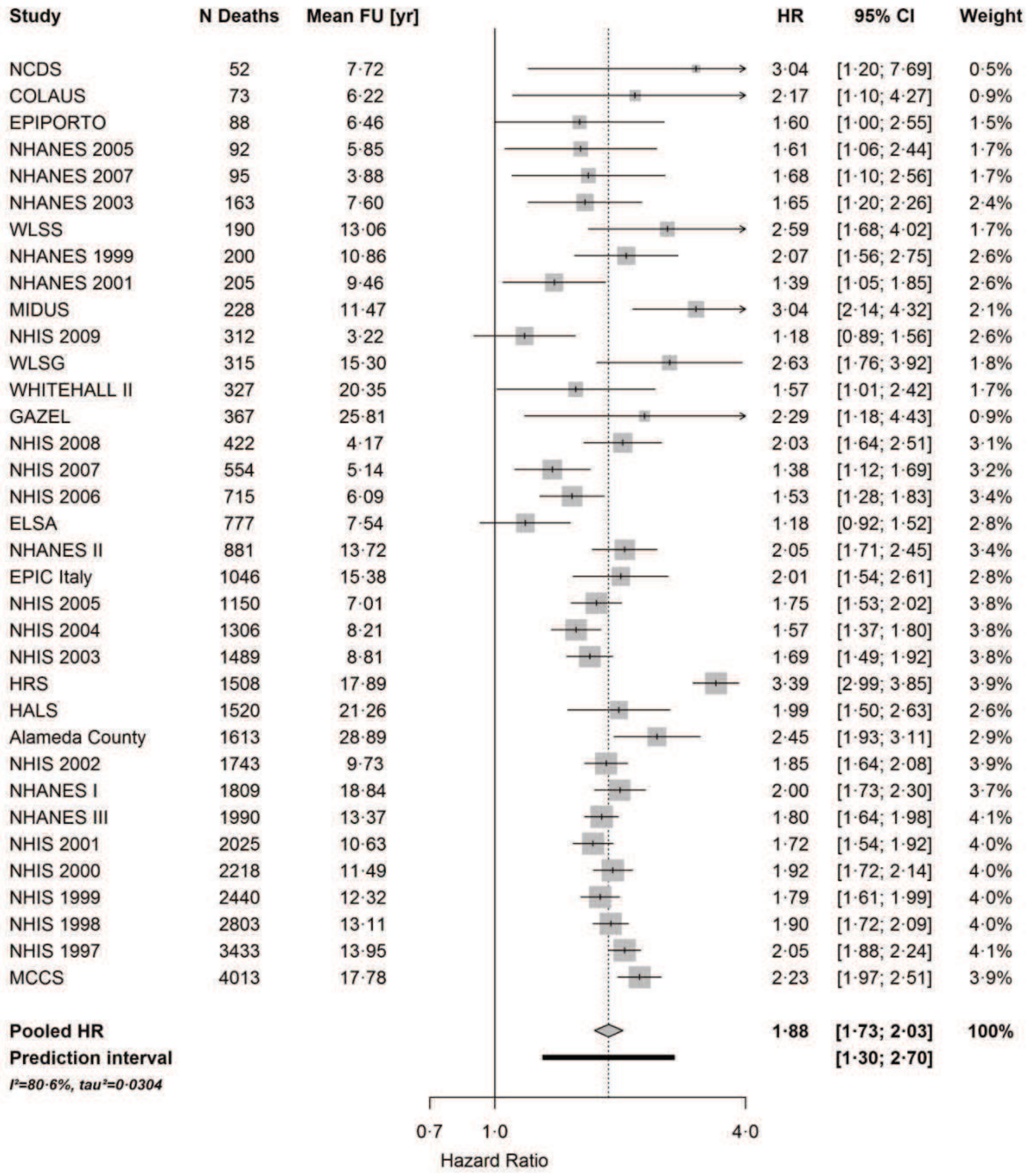

Pooled HR is represented with a gray diamond and the 95% prediction interval with a black bar. I² statistic is the percentage of between study heterogeneity; tau² statistics measures the inter-study variance. The prediction interval provides a predicted range for the true association between diabetes and all-cause mortality.

Age-, marital status- and race/ethnicity-adjusted hazard ratios for all-cause mortality in physically inactive compared to physically active participants – MEN

Study	N Deaths	Mean FU [yr]	HR	95% CI	Weight
COLAUS	136	6·13	1·58	[1·12; 2·21]	1·7%
EPIPORTO	140	6·83	1·54	[0·78; 3·05]	0·6%
NHANES 2007	192	3·86	2·52	[1·75; 3·64]	1·6%
NHANES 2005	239	5·73	1·81	[1·40; 2·35]	2·3%
MIDUS	257	11·41	1·89	[1·48; 2·42]	2·3%
WLSS	260	12·78	1·74	[1·28; 2·38]	1·9%
NHIS 2009	326	3·20	2·16	[1·70; 2·75]	2·4%
WLSG	367	15·08	1·56	[1·23; 1·99]	2·4%
NHIS 2008	397	4·16	2·20	[1·76; 2·75]	2·5%
NHANES 2003	399	7·39	1·59	[1·30; 1·95]	2·7%
NHANES 2001	502	9·10	1·80	[1·50; 2·17]	2·8%
NHIS 2007	514	5·10	2·01	[1·65; 2·44]	2·7%
NHANES 1999	517	10·48	1·46	[1·22; 1·75]	2·9%
NHIS 2006	661	6·05	2·00	[1·68; 2·38]	3·0%
WHITEHALL II	708	20·40	1·18	[0·97; 1·44]	2·7%
EPIC Italy	730	16·01	1·31	[1·10; 1·56]	2·9%
ELSA	817	7·33	2·11	[1·83; 2·43]	3·2%
NHIS 2005	1027	6·95	1·68	[1·47; 1·92]	3·3%
NHIS 2004	1162	8·15	1·88	[1·65; 2·13]	3·3%
NHIS 2003	1222	8·77	1·63	[1·44; 1·83]	3·4%
NHANES II	1247	12·80	1·40	[1·25; 1·57]	3·4%
HALS	1365	20·30	1·37	[1·21; 1·56]	3·3%
NHIS 2002	1422	9·64	1·61	[1·44; 1·80]	3·4%
GAZEL	1469	25·53	1·38	[1·24; 1·53]	3·5%
Alameda County	1525	27·56	1·50	[1·32; 1·71]	3·3%
NHIS 2001	1712	10·49	1·74	[1·57; 1·93]	3·5%
NHIS 2000	1866	11·36	1·58	[1·44; 1·74]	3·5%
NHIS 1999	2006	12·16	1·53	[1·39; 1·68]	3·6%
HRS	2204	16·33	1·60	[1·45; 1·75]	3·6%
NHIS 1998	2387	12·95	1·37	[1·26; 1·49]	3·6%
NHANES I	2415	16·70	1·32	[1·22; 1·43]	3·6%
NHANES III	2435	12·96	1·56	[1·43; 1·71]	3·6%
NHIS 1997	2693	13·82	1·51	[1·40; 1·64]	3·6%
MCCS	4475	17·01	1·08	[1·00; 1·16]	3·7%
**Pooled HR**			**1·60**	**[1·50; 1·70]**	**100%**
**Prediction interval**				**[1·16; 2·21]**	

I^2=85·1%, tau^2=0·0241

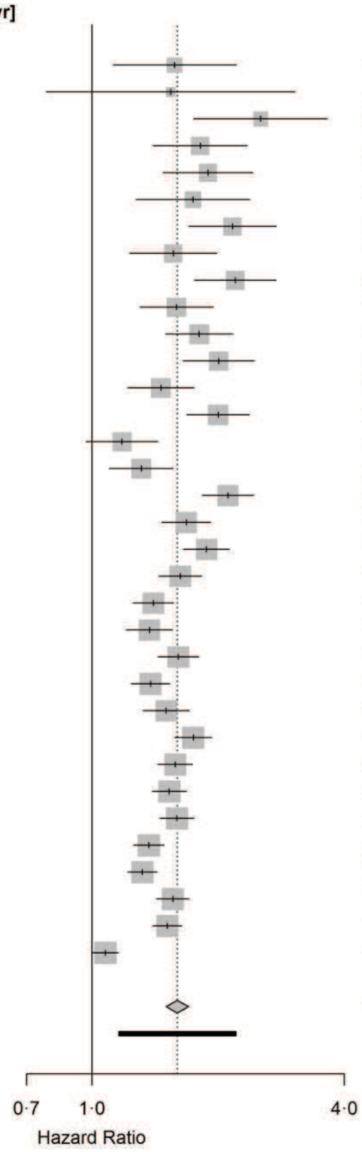

Pooled HR is represented with a gray diamond and the 95% prediction interval with a black bar. I² statistic is the percentage of between study heterogeneity; tau² statistics measures the inter-study variance. The prediction interval provides a predicted range for the true association between physical inactivity and all-cause mortality.

Age-, marital status- and race/ethnicity-adjusted hazard ratios for all-cause mortality in physically active compared to physically active participants – WOMEN

Study	N Deaths	Mean FU [yr]	HR	95% CI	Weight
COLAUS	73	6·22	1·97	[1·25; 3·13]	1·2%
EPIPORTO	75	6·44	1·31	[0·52; 3·29]	0·4%
NHANES 2007	159	3·91	1·77	[1·16; 2·71]	1·3%
NHANES 2005	184	5·83	1·61	[1·19; 2·18]	1·9%
WLSS	196	13·04	1·58	[1·15; 2·18]	1·8%
MIDUS	228	11·47	1·45	[1·10; 1·90]	2·2%
GAZEL	279	25·93	1·38	[1·09; 1·74]	2·4%
NHIS 2009	304	3·22	1·90	[1·45; 2·49]	2·2%
WLSG	312	15·30	1·58	[1·24; 2·01]	2·4%
WHITEHALL II	328	20·34	1·04	[0·83; 1·30]	2·5%
NHANES 2003	328	7·60	1·82	[1·42; 2·33]	2·3%
NHIS 2008	410	4·17	1·99	[1·56; 2·52]	2·4%
NHANES 1999	420	10·86	1·91	[1·51; 2·43]	2·4%
NHANES 2001	451	9·37	2·09	[1·68; 2·60]	2·6%
NHIS 2007	537	5·14	1·87	[1·52; 2·31]	2·6%
NHIS 2006	699	6·08	1·95	[1·63; 2·35]	2·9%
ELSA	751	7·57	1·85	[1·58; 2·16]	3·1%
NHANES II	881	13·72	1·32	[1·15; 1·51]	3·3%
EPIC Italy	1047	15·38	1·16	[1·03; 1·31]	3·4%
NHIS 2005	1110	7·01	1·85	[1·61; 2·14]	3·2%
NHIS 2004	1277	8·21	1·80	[1·58; 2·05]	3·3%
NHIS 2003	1455	8·81	1·67	[1·48; 1·88]	3·4%
HRS	1488	17·90	1·62	[1·45; 1·80]	3·5%
HALS	1520	21·26	1·61	[1·40; 1·84]	3·3%
Alameda County	1561	28·98	1·36	[1·22; 1·52]	3·5%
NHIS 2002	1699	9·73	1·71	[1·53; 1·92]	3·5%
NHIS 2001	1958	10·63	1·81	[1·63; 2·02]	3·5%
NHANES I	2064	19·03	1·25	[1·14; 1·36]	3·6%
NHANES III	2138	13·37	1·56	[1·43; 1·71]	3·7%
NHIS 2000	2171	11·48	1·74	[1·57; 1·92]	3·6%
NHIS 1999	2395	12·31	1·37	[1·25; 1·50]	3·6%
NHIS 1998	2760	13·11	1·63	[1·49; 1·78]	3·7%
NHIS 1997	3384	13·95	1·56	[1·44; 1·68]	3·7%
MCCS	4013	17·78	1·15	[1·07; 1·24]	3·7%
E3N	6698	16·21	1·33	[1·26; 1·40]	3·8%
**Pooled HR**			**1·58**	**[1·48; 1·67]**	**100%**
**Prediction interval**				**[1·15; 2·16]**	

I²=84·8%, tau²=0·0229

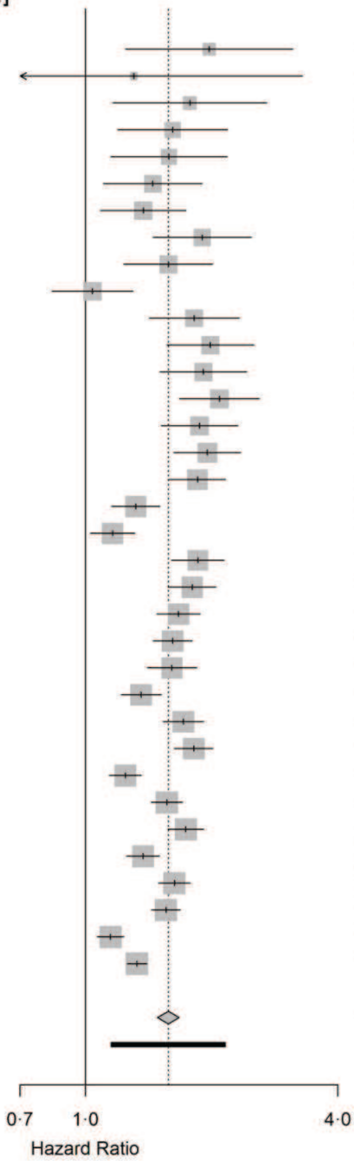

Pooled HR is represented with a gray diamond and the 95% prediction interval with a black bar. I² statistic is the percentage of between study heterogeneity; tau² statistics measures the inter-study variance. The prediction interval provides a predicted range for the true association between physical inactivity and all-cause mortality.

Age-, marital status- and race/ethnicity-adjusted hazard ratios for all-cause mortality in participants with high alcohol intake compared to those with moderate intake – MEN

Study	N Deaths	Mean FU [yr]	HR	95% CI	Weight
COLAUS	136	6·14	1·64	[1·13; 2·40]	2·4%
EPIPORTO	139	6·84	1·07	[0·73; 1·58]	2·3%
NCDS	140	7·45	1·50	[1·01; 2·23]	2·3%
NHANES 2007	162	3·87	1·11	[0·55; 2·25]	1·0%
NHANES 2005	195	5·72	1·37	[0·70; 2·67]	1·1%
NHIS 2009	201	3·22	2·32	[1·31; 4·10]	1·4%
MIDUS	254	11·41	0·85	[0·53; 1·36]	1·8%
WLSG	263	15·09	1·52	[0·94; 2·46]	1·8%
NHIS 2008	264	4·18	2·04	[1·27; 3·28]	1·8%
NHANES 2003	320	7·36	1·80	[1·19; 2·72]	2·1%
NHIS 2007	323	5·14	1·84	[1·09; 3·11]	1·6%
WLSS	342	12·75	1·40	[0·81; 2·41]	1·5%
NHANES 2001	402	9·11	1·18	[0·81; 1·73]	2·4%
NHANES 1999	403	10·50	1·64	[1·08; 2·49]	2·1%
NHIS 2006	450	6·09	1·58	[0·97; 2·57]	1·7%
NHIS 2005	676	7·02	1·05	[0·68; 1·64]	2·0%
WHITEHALL II	708	20·40	1·70	[1·41; 2·05]	3·9%
EPIC Italy	727	16·01	1·04	[0·88; 1·24]	4·1%
NHIS 2004	765	8·24	2·45	[1·82; 3·28]	3·0%
NHIS 2003	831	8·87	1·85	[1·36; 2·53]	2·9%
ELSA	843	7·29	1·06	[0·86; 1·32]	3·7%
NHIS 2002	937	9·79	2·19	[1·68; 2·85]	3·3%
NHIS 2001	1131	10·67	1·94	[1·48; 2·55]	3·2%
NHANES II	1244	12·80	1·35	[0·95; 1·92]	2·6%
NHIS 2000	1273	11·54	2·02	[1·57; 2·59]	3·4%
NHIS 1999	1359	12·40	1·78	[1·41; 2·26]	3·5%
HALS	1364	20·29	1·23	[1·06; 1·43]	4·3%
GAZEL	1387	25·58	1·51	[1·35; 1·70]	4·5%
Alameda County	1472	27·60	1·24	[1·02; 1·52]	3·8%
NHIS 1998	1585	13·25	1·75	[1·40; 2·19]	3·6%
NHIS 1997	1830	14·13	1·64	[1·32; 2·04]	3·7%
NHANES III	2148	13·12	1·52	[1·25; 1·86]	3·8%
NHANES I	2168	16·74	1·31	[1·12; 1·54]	4·2%
HRS	2245	16·30	1·66	[1·45; 1·90]	4·4%
MCCS	4464	17·02	1·22	[1·13; 1·31]	4·8%
**Pooled HR**			**1·50**	**[1·38; 1·64]**	**100%**
**Prediction interval**				**[1·03; 2·20]**	

I²=71·3%, tau²=0·0332

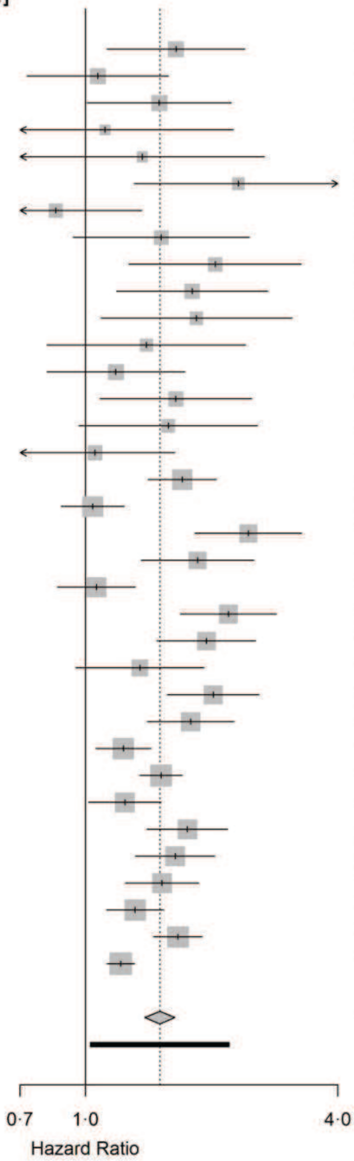

Pooled HR is represented with a gray diamond and the 95% prediction interval with a black bar. I² statistic is the percentage of between study heterogeneity; tau² statistics measures the inter-study variance. The prediction interval provides a predicted range for the true association between high alcohol intake and all-cause mortality.

Age-, marital status- and race/ethnicity-adjusted hazard ratios for all-cause mortality in participants with high alcohol intake compared to those with moderate intake – WOMEN

Study	N Deaths	Mean FU [yr]	HR	95% CI	Weight
COLAUS	73	6·22	1·66	[0·89; 3·09]	2·2%
EPIPORTO	74	6·43	1·59	[0·71; 3·55]	1·6%
NCDS	93	7·72	1·28	[0·71; 2·30]	2·4%
NHANES 2007	122	3·92	1·20	[0·28; 5·15]	0·6%
NHANES 2005	132	5·84	4·36	[2·05; 9·28]	1·7%
WLSG	204	15·30	1·71	[0·97; 3·00]	2·5%
NHIS 2009	205	3·22	1·00	[0·14; 7·21]	0·3%
MIDUS	226	11·47	1·91	[1·08; 3·39]	2·5%
WLSS	226	13·09	1·72	[0·80; 3·71]	1·7%
NHANES 2003	246	7·62	1·34	[0·53; 3·35]	1·3%
GAZEL	264	25·94	1·76	[1·21; 2·55]	3·6%
NHIS 2008	267	4·19	0·66	[0·09; 4·70]	0·3%
NHANES 1999	310	10·89	1·17	[0·54; 2·54]	1·7%
NHANES 2001	316	9·46	1·18	[0·59; 2·34]	2·0%
WHITEHALL II	327	20·35	1·32	[0·91; 1·91]	3·6%
NHIS 2007	383	5·16	2·82	[1·32; 6·04]	1·7%
NHIS 2006	506	6·11	3·48	[1·78; 6·80]	2·0%
ELSA	777	7·54	0·97	[0·70; 1·34]	4·0%
NHIS 2005	794	7·05	1·72	[0·85; 3·48]	1·9%
NHANES II	875	13·73	1·57	[0·81; 3·05]	2·1%
NHIS 2004	934	8·25	3·08	[1·77; 5·37]	2·5%
EPIC Italy	1047	15·38	1·38	[1·11; 1·72]	4·7%
NHIS 2003	1078	8·87	1·98	[1·14; 3·44]	2·6%
NHIS 2002	1215	9·82	1·45	[0·75; 2·80]	2·1%
NHIS 2001	1470	10·72	3·21	[2·22; 4·63]	3·6%
HRS	1508	17·89	2·27	[1·69; 3·05]	4·1%
HALS	1520	21·26	0·97	[0·73; 1·28]	4·3%
Alameda County	1532	28·93	1·34	[1·02; 1·77]	4·3%
NHIS 2000	1611	11·60	2·10	[1·31; 3·36]	3·0%
NHANES I	1755	18·98	1·47	[1·10; 1·97]	4·2%
NHIS 1999	1809	12·43	1·86	[1·26; 2·75]	3·5%
NHANES III	1832	13·57	2·45	[1·61; 3·71]	3·3%
NHIS 1998	2009	13·27	2·38	[1·62; 3·49]	3·5%
NHIS 1997	2475	14·14	2·61	[1·85; 3·67]	3·8%
MCCS	4011	17·78	1·27	[1·14; 1·41]	5·3%
E3N	5638	17·22	1·21	[1·13; 1·29]	5·4%
**Pooled HR**			**1·69**	**[1·49; 1·92]**	**100%**
**Prediction interval**				**[0·98; 2·91]**	

I²=73%, tau²=0·0678

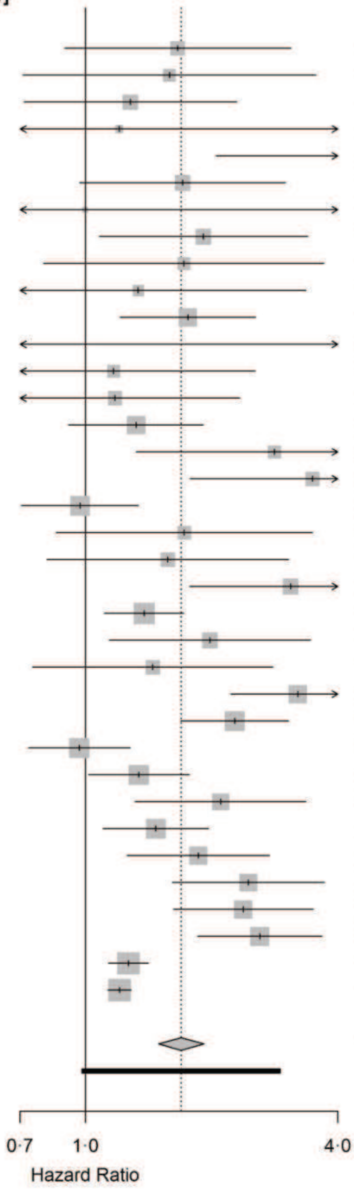

Pooled HR is represented with a gray diamond and the 95% prediction interval with a black bar. I² statistic is the percentage of between study heterogeneity; tau² statistics measures the inter-study variance. The prediction interval provides a predicted range for the true association between high alcohol intake and all-cause mortality.

Age-, marital status- and race/ethnicity-adjusted hazard ratios for all-cause mortality in hypertensive compared to normotensive participants – MEN

Study	N Deaths	Mean FU [yr]	HR	95% CI	Weight
COLAUS	136	6·14	1·02	[0·70; 1·48]	1·0%
EPIPORTO	139	6·84	1·63	[1·11; 2·38]	0·9%
NCDS	164	7·45	1·23	[0·89; 1·71]	1·2%
NHANES 2007	192	3·86	1·15	[0·81; 1·62]	1·1%
NHANES 2005	239	5·73	1·23	[0·90; 1·69]	1·3%
WLSS	254	12·81	1·13	[0·87; 1·47]	1·7%
MIDUS	258	11·41	1·47	[1·13; 1·91]	1·7%
NHIS 2009	333	3·20	1·24	[0·99; 1·55]	2·0%
WLSG	370	15·08	1·89	[1·53; 2·34]	2·1%
NHANES 2003	399	7·39	1·25	[0·97; 1·62]	1·7%
NHIS 2008	415	4·16	1·29	[1·06; 1·59]	2·3%
NHANES 2001	502	9·10	1·38	[1·10; 1·74]	1·9%
NHANES 1999	517	10·48	1·33	[1·05; 1·68]	1·9%
NHIS 2007	532	5·11	1·23	[1·03; 1·47]	2·6%
NHIS 2006	681	6·05	1·45	[1·24; 1·70]	2·9%
WHITEHALL II	708	20·40	1·14	[0·97; 1·34]	2·9%
EPIC Italy	727	16·01	1·23	[1·06; 1·42]	3·1%
ELSA	843	7·29	0·89	[0·77; 1·02]	3·2%
NHIS 2005	1052	6·95	1·30	[1·14; 1·47]	3·5%
NHIS 2004	1197	8·15	1·30	[1·16; 1·46]	3·6%
NHIS 2003	1245	8·78	1·26	[1·12; 1·41]	3·6%
NHANES II	1247	12·80	1·17	[1·02; 1·34]	3·3%
HALS	1365	20·30	1·19	[1·04; 1·36]	3·3%
GAZEL	1387	25·58	1·56	[1·37; 1·78]	3·3%
NHIS 2002	1469	9·64	1·29	[1·16; 1·43]	3·8%
Alameda County	1560	27·48	1·59	[1·37; 1·85]	3·0%
NHIS 2001	1776	10·49	1·37	[1·25; 1·51]	4·0%
NHIS 2000	1917	11·37	1·27	[1·16; 1·40]	4·0%
NHIS 1999	2063	12·17	1·34	[1·23; 1·47]	4·1%
HRS	2245	16·30	1·51	[1·38; 1·64]	4·2%
NHANES I	2410	16·70	1·30	[1·17; 1·44]	3·9%
NHIS 1998	2428	12·96	1·34	[1·24; 1·46]	4·2%
NHANES III	2442	12·97	1·25	[1·13; 1·37]	4·0%
NHIS 1997	2764	13·82	1·36	[1·26; 1·47]	4·3%
MCCS	4464	17·02	1·15	[1·08; 1·23]	4·5%
**Pooled HR**			**1·30**	**[1·24; 1·36]**	**100%**
**Prediction interval**				**[1·07; 1·57]**	

I²=66·4%, tau²=0·0085

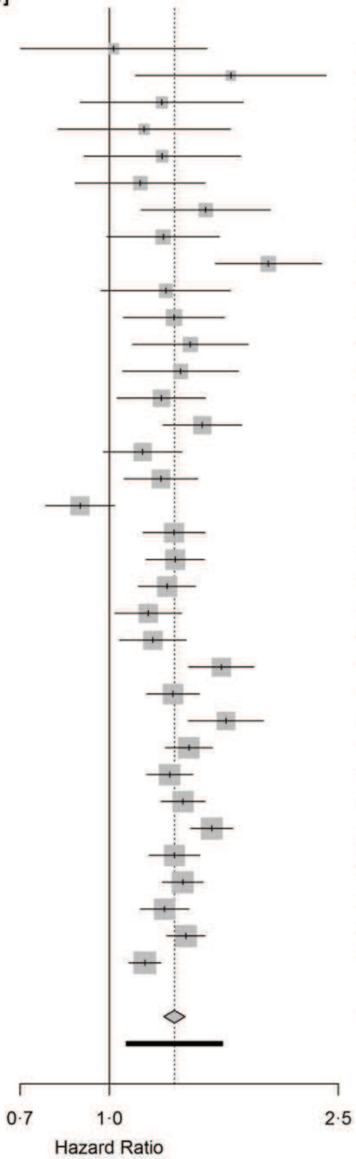

Pooled HR is represented with a gray diamond and the 95% prediction interval with a black bar. I² statistic is the percentage of between study heterogeneity; tau² statistics measures the inter-study variance. The prediction interval provides a predicted range for the true association between hypertension and all-cause mortality.

Age-, marital status- and race/ethnicity-adjusted hazard ratios for all-cause mortality in hypertensive compared to normotensive participants – WOMEN

Study	N Deaths	Mean FU [yr]	HR	95% CI	Weight
COLAUS	73	6·22	1·00	[0·62; 1·62]	1·0%
EPIPORTO	74	6·43	1·01	[0·57; 1·80]	0·7%
NCDS	118	7·72	1·53	[0·99; 2·37]	1·1%
NHANES 2007	159	3·91	0·91	[0·58; 1·41]	1·1%
NHANES 2005	184	5·83	1·34	[0·89; 2·03]	1·2%
WLSS	190	13·06	1·48	[1·10; 1·99]	1·9%
MIDUS	230	11·46	2·04	[1·56; 2·67]	2·1%
GAZEL	264	25·94	1·27	[0·90; 1·79]	1·6%
NHIS 2009	312	3·22	0·91	[0·72; 1·15]	2·4%
WLSG	315	15·30	1·39	[1·08; 1·79]	2·2%
WHITEHALL II	327	20·35	1·11	[0·87; 1·42]	2·3%
NHANES 2003	328	7·60	1·41	[0·97; 2·04]	1·4%
NHIS 2008	421	4·18	1·40	[1·13; 1·74]	2·5%
NHANES 1999	421	10·85	1·06	[0·80; 1·42]	1·9%
NHANES 2001	451	9·37	1·01	[0·76; 1·35]	1·9%
NHIS 2007	554	5·14	1·23	[1·03; 1·47]	2·9%
NHIS 2006	713	6·09	1·21	[1·03; 1·41]	3·1%
ELSA	777	7·54	0·96	[0·82; 1·11]	3·2%
NHANES II	873	13·73	1·25	[1·06; 1·47]	3·0%
EPIC Italy	1047	15·38	1·21	[1·07; 1·38]	3·4%
NHIS 2005	1150	7·01	1·24	[1·09; 1·40]	3·5%
NHIS 2004	1301	8·21	1·17	[1·05; 1·32]	3·5%
NHIS 2003	1487	8·82	1·25	[1·12; 1·39]	3·6%
HRS	1508	17·89	1·83	[1·65; 2·03]	3·6%
HALS	1520	21·26	1·32	[1·16; 1·51]	3·4%
Alameda County	1610	28·89	1·52	[1·35; 1·71]	3·5%
NHIS 2002	1737	9·73	1·25	[1·13; 1·38]	3·7%
NHIS 2001	2023	10·63	1·25	[1·14; 1·37]	3·8%
NHANES I	2053	19·04	1·51	[1·34; 1·72]	3·4%
NHANES III	2143	13·37	1·32	[1·17; 1·48]	3·5%
NHIS 2000	2216	11·49	1·43	[1·31; 1·56]	3·8%
NHIS 1999	2430	12·32	1·40	[1·29; 1·52]	3·8%
NHIS 1998	2803	13·11	1·49	[1·38; 1·61]	3·9%
NHIS 1997	3429	13·95	1·29	[1·21; 1·39]	3·9%
MCCS	4011	17·78	1·24	[1·16; 1·32]	3·9%
E3N	5638	17·22	1·03	[0·97; 1·09]	4·0%
**Pooled HR**			**1·28**	**[1·21; 1·36]**	**100%**
**Prediction interval**				**[0·97; 1·70]**	

I²=81·8%, tau²=0·0186

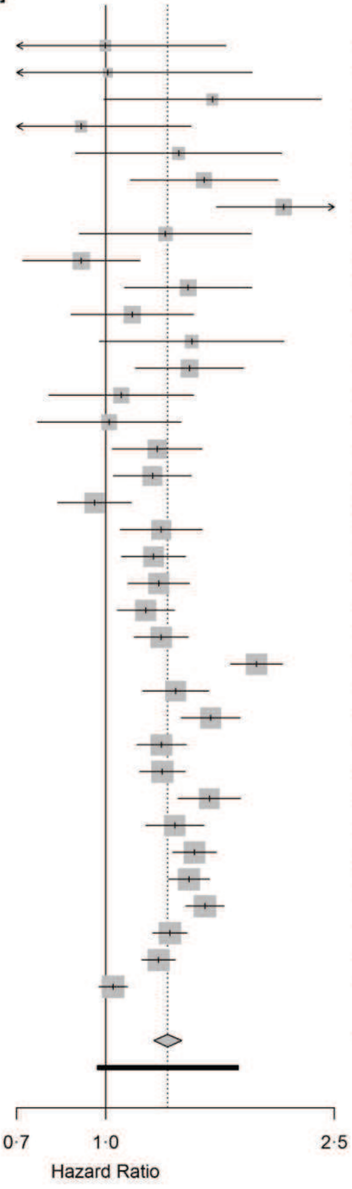

Pooled HR is represented with a gray diamond and the 95% prediction interval with a black bar. I² statistic is the percentage of between study heterogeneity; tau² statistics measures the inter-study variance. The prediction interval provides a predicted range for the true association between hypertension and all-cause mortality.

Age-, marital status- and race/ethnicity-adjusted hazard ratios for all-cause mortality in obese compared to normal BMI participants – MEN

Study	N Deaths	Mean FU [yr]	HR	95% CI	Weight
COLAUS	136	6·14	0·87	[0·57; 1·34]	0·9%
EPIPORTO	139	6·80	1·25	[0·76; 2·05]	0·7%
NCDS	159	7·45	1·01	[0·67; 1·52]	0·9%
NHANES 2007	160	3·87	0·65	[0·43; 0·97]	1·0%
NHANES 2005	201	5·77	0·72	[0·50; 1·04]	1·1%
MIDUS	241	11·42	0·84	[0·59; 1·20]	1·2%
WLSS	256	12·80	1·27	[0·90; 1·81]	1·1%
NHIS 2009	319	3·20	0·81	[0·61; 1·07]	1·5%
NHANES 2003	327	7·46	0·94	[0·71; 1·24]	1·5%
NHANES 2001	337	9·37	0·66	[0·50; 0·89]	1·4%
WLSG	367	15·08	1·43	[1·08; 1·89]	1·5%
NHIS 2008	385	4·16	0·82	[0·63; 1·07]	1·6%
NHANES 1999	420	10·66	0·82	[0·64; 1·07]	1·6%
NHIS 2007	502	5·11	0·74	[0·59; 0·93]	1·8%
ELSA	625	7·49	0·88	[0·71; 1·08]	1·9%
NHIS 2006	639	6·06	0·83	[0·68; 1·02]	2·0%
WHITEHALL II	655	20·44	1·95	[1·51; 2·53]	1·6%
EPIC Italy	753	16·01	1·56	[1·27; 1·91]	2·0%
NHIS 2005	1001	6·96	0·89	[0·76; 1·05]	2·3%
HALS	1081	20·57	1·30	[1·06; 1·59]	2·0%
NHIS 2004	1141	8·16	0·89	[0·76; 1·04]	2·4%
NHIS 2003	1201	8·78	0·83	[0·71; 0·97]	2·4%
NHANES II	1206	12·84	0·89	[0·75; 1·05]	2·2%
NHIS 2002	1397	9·66	0·82	[0·71; 0·94]	2·5%
GAZEL	1511	25·49	1·77	[1·47; 2·13]	2·1%
Alameda County	1539	27·47	1·43	[1·17; 1·75]	2·0%
NHIS 2001	1709	10·51	0·89	[0·79; 1·02]	2·6%
NHIS 2000	1861	11·38	0·88	[0·78; 1·00]	2·6%
NHIS 1999	1995	12·18	0·94	[0·83; 1·06]	2·6%
HRS	2217	16·35	1·12	[0·99; 1·25]	2·7%
NHANES I	2323	16·81	1·24	[1·10; 1·40]	2·6%
NHANES III	2356	13·03	0·85	[0·76; 0·95]	2·7%
NHIS 1998	2366	12·98	0·95	[0·85; 1·06]	2·7%
NHIS 1997	2701	13·84	0·94	[0·85; 1·05]	2·7%
NHIS 1996	3329	14·64	1·08	[0·98; 1·19]	2·8%
MCCS	4455	17·02	1·26	[1·16; 1·37]	2·9%
NHIS 1995	6092	15·36	1·02	[0·95; 1·09]	2·9%
NHIS 1986	6334	21·82	1·28	[1·18; 1·38]	2·9%
NHIS 1993	7649	16·93	1·10	[1·03; 1·17]	3·0%
NHIS 1994	7727	16·11	1·10	[1·03; 1·17]	3·0%
NHIS 1992	9278	18·47	1·11	[1·05; 1·18]	3·0%
NHIS 1991	9409	18·40	1·15	[1·09; 1·22]	3·0%
NHIS 1990	9860	19·17	1·17	[1·10; 1·24]	3·0%
NHIS 1989	10272	19·83	1·13	[1·07; 1·20]	3·0%
NHIS 1988	11348	20·50	1·19	[1·13; 1·26]	3·0%
NHIS 1987	11903	21·14	1·23	[1·17; 1·30]	3·0%
**Pooled HR**			**1·04**	**[0·98; 1·11]**	**100%**
**Prediction interval**				**[0·79; 1·38]**	

I^2=85·6%, tau^2=0·0184

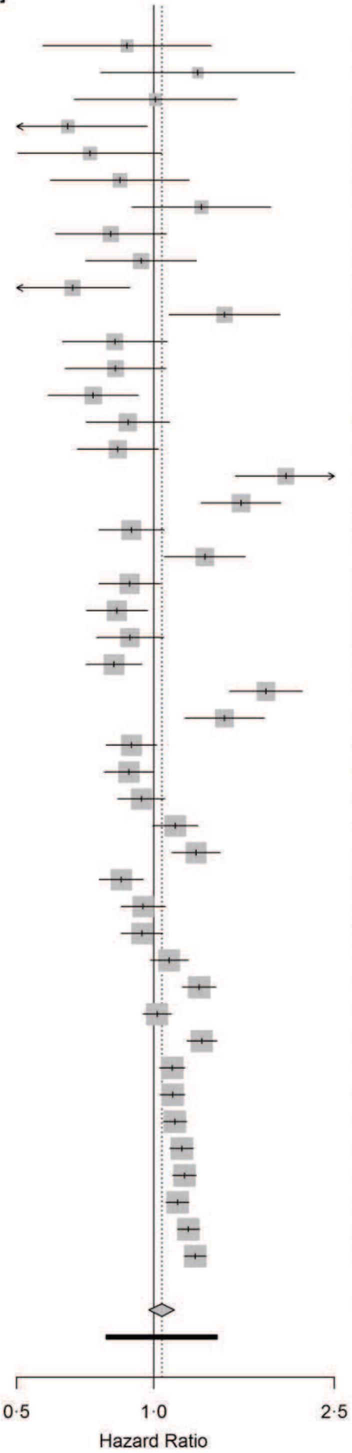

Pooled HR is represented with a gray diamond and the 95% prediction interval with a black bar. I² statistic is the percentage of between study heterogeneity; tau² statistics measures the inter-study variance. The prediction interval provides a predicted range for the true association between obesity and all-cause mortality.

Age-, marital status- and race/ethnicity-adjusted hazard ratios for all-cause mortality in obese compared to normal BMI participants – WOMEN

Study	N Deaths	Mean FU [yr]	HR	95% CI	Weight
COLAUS	71	6·22	1·04	[0·54; 2·00]	0·4%
EPIPORTO	82	6·45	1·89	[1·04; 3·45]	0·4%
NCDS	115	7·72	0·95	[0·60; 1·49]	0·7%
NHANES 2007	123	3·93	0·66	[0·43; 1·01]	0·8%
NHANES 2005	142	5·87	0·72	[0·48; 1·07]	0·8%
WLSS	187	13·05	1·47	[1·02; 2·10]	1·0%
MIDUS	210	11·46	1·12	[0·80; 1·57]	1·1%
NHIS 2009	263	3·22	0·77	[0·57; 1·04]	1·2%
NHANES 2003	264	7·66	1·06	[0·78; 1·45]	1·2%
NHANES 2001	266	9·61	1·10	[0·81; 1·50]	1·2%
GAZEL	271	25·92	2·44	[1·60; 3·73]	0·8%
WHITEHALL II	287	20·40	1·59	[1·18; 2·16]	1·2%
WLSG	299	15·31	1·50	[1·13; 1·99]	1·4%
NHANES 1999	305	11·08	0·90	[0·68; 1·20]	1·3%
NHIS 2008	365	4·18	0·89	[0·69; 1·16]	1·5%
NHIS 2007	476	5·14	0·74	[0·58; 0·93]	1·7%
ELSA	535	7·75	1·04	[0·84; 1·30]	1·8%
NHIS 2006	637	6·09	0·92	[0·75; 1·11]	2·0%
NHANES II	840	13·75	1·05	[0·88; 1·25]	2·1%
NHIS 2005	1020	7·02	1·05	[0·90; 1·22]	2·3%
HALS	1047	21·62	1·43	[1·21; 1·70]	2·2%
EPIC Italy	1060	15·39	1·49	[1·27; 1·74]	2·3%
NHIS 2004	1180	8·22	0·92	[0·79; 1·06]	2·4%
NHIS 2003	1330	8·83	0·91	[0·79; 1·04]	2·4%
HRS	1448	17·95	1·50	[1·32; 1·71]	2·5%
Alameda County	1507	28·98	1·20	[1·01; 1·42]	2·2%
NHIS 2002	1563	9·75	0·96	[0·85; 1·09]	2·5%
NHIS 2001	1837	10·66	1·04	[0·92; 1·16]	2·6%
NHANES I	1978	19·05	1·17	[1·04; 1·30]	2·7%
NHIS 2000	1981	11·52	1·12	[1·01; 1·25]	2·7%
NHANES III	2024	13·42	1·06	[0·95; 1·18]	2·7%
NHIS 1999	2223	12·35	1·06	[0·95; 1·18]	2·7%
NHIS 1998	2574	13·15	1·04	[0·94; 1·15]	2·8%
NHIS 1997	3123	14·01	1·08	[0·98; 1·18]	2·9%
NHIS 1996	3256	14·81	1·15	[1·05; 1·26]	2·9%
MCCS	3950	17·80	1·27	[1·17; 1·38]	2·9%
NHIS 1995	5923	15·63	1·16	[1·08; 1·24]	3·0%
NHIS 1986	6284	22·45	1·39	[1·30; 1·48]	3·0%
NHIS 1993	7536	17·21	1·17	[1·11; 1·25]	3·1%
NHIS 1994	7748	16·32	1·26	[1·19; 1·34]	3·1%
E3N	8016	16·24	1·58	[1·43; 1·73]	2·8%
NHIS 1992	9256	18·82	1·23	[1·17; 1·30]	3·1%
NHIS 1991	9280	18·79	1·33	[1·26; 1·40]	3·1%
NHIS 1990	9929	19·54	1·27	[1·21; 1·34]	3·1%
NHIS 1989	10354	20·25	1·34	[1·27; 1·41]	3·1%
NHIS 1988	11534	20·97	1·35	[1·28; 1·41]	3·1%
NHIS 1987	11981	21·71	1·37	[1·31; 1·44]	3·1%
**Pooled HR**			**1·17**	**[1·10; 1·24]**	**100%**
**Prediction interval**				**[0·91; 1·50]**	

I^2=84·9%, tau^2=0·0147

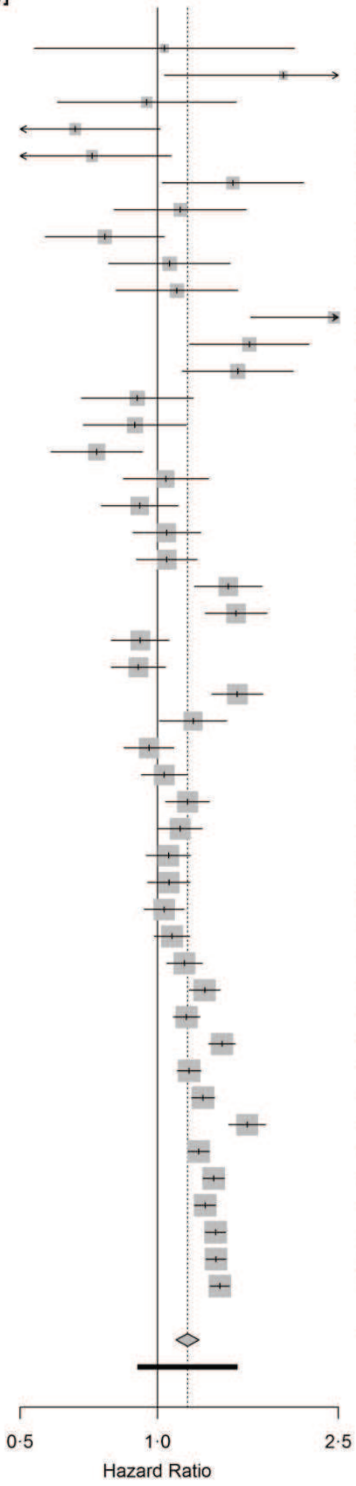

Pooled HR is represented with a gray diamond and the 95% prediction interval with a black bar. I² statistic is the percentage of between study heterogeneity; tau² statistics measures the inter-study variance. The prediction interval provides a predicted range for the true association between obesity and all-cause mortality.
